# Chemical Constituents of the Endophytic Fungus *Penicillium steckii* HJT-A-10 from *Rhodiola tibetica* and Their Anti-Inflammatory Activities

**DOI:** 10.3390/molecules31173109

**Published:** 2026-09-04

**Authors:** Yujie Lu, Haoyu Xiang, Yanjie Xu, Xinge Yu, Janar Jenis, Baomin Feng, Dongliang Xiao, Xuan Lu

**Affiliations:** 1 College of Life and Health, Dalian University, Dalian 116622, China; luyujie1227@126.com (Y.L.); xianghaoyuu@163.com (H.X.); 13945861421@163.com (Y.X.); yuxinge_1027@163.com (X.Y.); fbmdlu@163.com (B.F.); 2 Dalian Institute of Marine Traditional Chinese Medicine, China Academy of Chinese Medical Sciences, Dalian University, Dalian 116622, China; 3 Research Center for Medicinal Plants, Al-Farabi Kazakh National University, Almaty 050040, Kazakhstan; janarjenis@kaznu.kz; 4 Research Institute for Natural Products & Technology, Almaty 050040, Kazakhstan; 5 State Key Laboratory for Quality Ensurance and Sustainable Use of Dao-di Herbs, Beijing 100700, China

**Keywords:** *Penicillium steckii*, polyketides, alkaloids, nitric oxide inhibition

## Abstract

Chemical examination of *Penicillium steckii* HJT-A-10, an endophytic fungus derived from *Rhodiola tibetica*, led to the isolation of ten metabolites. Structural elucidation by spectroscopic methods revealed that two of these were previously unreported compounds (**1** and **2**), though the absolute stereochemistry of **2** remained provisional; the other eight isolates (**3**–**10**) were identified as known compounds. All ten metabolites were tested for their capacity to inhibit nitric oxide (NO) production in RAW 264.7 macrophages stimulated with lipopolysaccharide (LPS). Compounds **1**–**4** and **7**–**10** exhibited marked inhibitory effects. Among the two new compounds, **2** displayed stronger activity, with an IC_50_ value of 19.01 µM. To explore the potential mechanisms of action, network pharmacology and molecular docking analyses were subsequently conducted to predict the relevant targets and signaling pathways.

## 1. Introduction

Endophytic fungi are recognized as prolific sources of structurally diverse and biologically active secondary metabolites [1,2,3]. Of particular interest are endophytes associated with medicinal plants, some of which produce metabolites that are structurally or functionally related, or even identical, to constituents of their hosts [4,5,6,7,8]. Cultivation of these microorganisms may therefore provide a sustainable approach to accessing valuable natural products while reducing dependence on limited wild plant resources [9,10,11,12].

*Rhodiola tibetica* is a cold-adapted medicinal species native to the Qinghai–Tibet Plateau [13]. The declining availability of wild *Rhodiola* populations, together with sustained medicinal demand, has prompted interest in the microorganisms associated with this plant. Our previous investigations of *Rhodiola* endophytes yielded polyketides, alkaloids, and other metabolites that promoted seed germination, inhibited SARS-CoV-2 replication, or attenuated inflammatory responses [13,14,15,16,17,18]. Collectively, these findings identify the endophytic community of *Rhodiola* as a promising source of chemically diverse bioactive metabolites.

**Figure 1:**
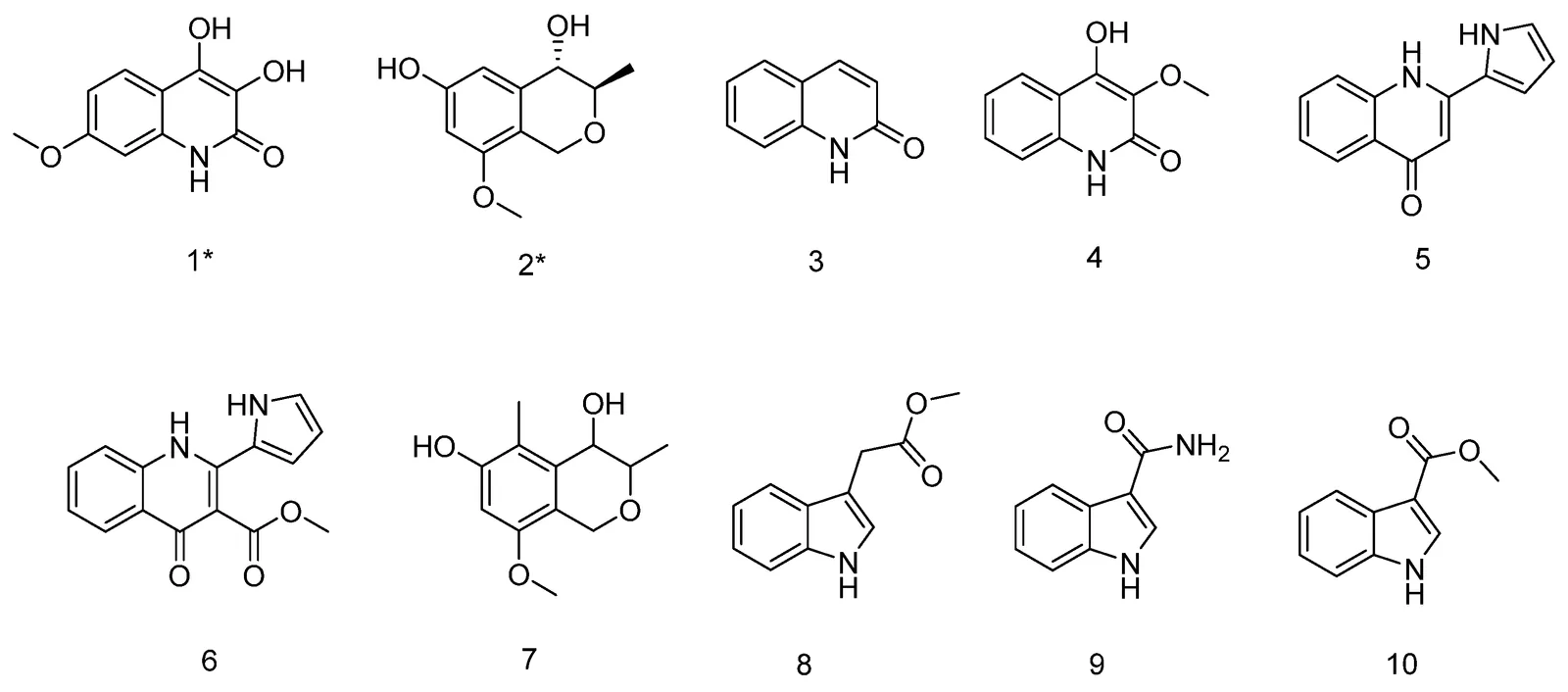
Chemical structures of metabolites **1**–**10** obtained from cultures of *Penicillium steckii* HJT-A-10 (* indicates a new compound).

In the present study, *Penicillium steckii* HJT-A-10, recovered from roots of *R. tibetica*, was selected for a chemistry-guided investigation. Fractionation of its culture extract furnished two new metabolites and eight known compounds (Figure 1), whose structures were assigned from spectroscopic evidence. The ten isolates were evaluated in an LPS-stimulated RAW 264.7 macrophage model, and compound **2** was further examined computationally by target-network analysis and molecular docking to identify plausible inflammation-related targets and pathways.

## 2. Results and Discussion

### 2.1. Isolation and Structural Characterization

**Figure 2:**
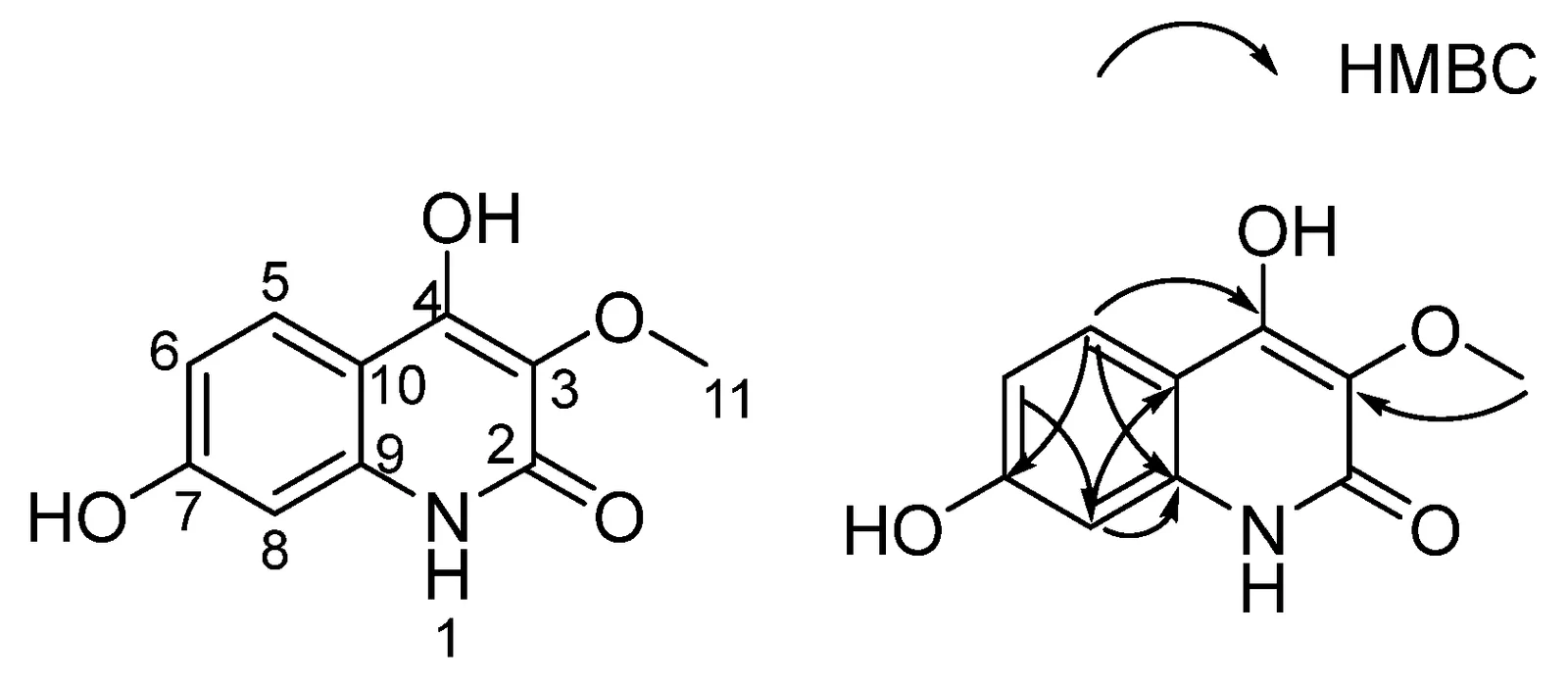
Structure of compound **1** and the diagnostic HMBC correlations supporting its assignment.

Compound **1** was obtained as a purple powder. Its sodium-adduct ion at *m*/*z* 230.0429 (calcd for C_10_H_9_NO_4_Na, 230.0424) established the molecular formula C_10_H_9_NO_4_, corresponding to seven degrees of unsaturation. The ^1^H NMR (Table 1) and HSQC spectra revealed an amide NH (*δ*_H_ 10.09, s), a phenolic OH (*δ*_H_ 11.15, s), a methoxy group (*δ*_H_ 3.69, s), and three aromatic methines consistent with a 1,2,4-trisubstituted ring [*δ*_H_ 7.56 (d, *J* = 8.5 Hz), 6.61 (dd, *J* = 8.5, 2.5 Hz), and 6.63 (d, *J* = 2.5 Hz)]. The ^13^C NMR spectrum contained ten resonances, including a methoxy carbon at *δ*_C_ 59.3, three protonated aromatic carbons at *δ*_C_ 123.9, 111.0, and 99.6, one carbonyl carbon at *δ*_C_ 160.2, two oxygenated nonprotonated sp^2^ carbons at *δ*_C_ 152.2 and 158.7, and three additional nonprotonated sp^2^ carbons at *δ*_C_ 137.4, 128.4, and 108.5. These data closely resembled those of 4-hydroxy-3-methoxy-2(1H)-quinolinone (**4**) [19] and supported the same 2-quinolinone framework. Whereas compound **4** displayed four aromatic proton signals, compound **1** showed only three together with an additional exchangeable proton, indicating that 1 was a hydroxylated analogue of **4** (Figure 2).

The HMBC correlation from the methoxy singlet (*δ*_H_ 3.69, H_3_-11) to C-3 (*δ*_C_ 128.4) placed the methoxy group at C-3. Comparison with compound **4**, together with the characteristic downfield shift of the second oxygenated aromatic carbon, located the additional hydroxy group at C-7. The constitution of **1** was therefore assigned as 4,7-dihydroxy-3-methoxyquinolin-2(1H)-one.

Compound **2** was obtained as a yellow oil. HRESIMS gave a sodium-adduct ion at *m*/*z* 233.0788 (calcd for C_11_H_14_O_4_Na, 233.0784), corresponding to C_11_H_14_O_4_ and five degrees of unsaturation. Its ^1^H and ^13^C NMR profiles (Table 2) were similar to those of the co-isolated isochromane **7** [20]. However, the aromatic methyl singlet of **7** was absent from the spectrum of **2**, while an additional aromatic proton appeared at *δ*_H_ 6.54. These differences identified **2** as a demethylated congener of **7**.

The aromatic substitution pattern was defined by HMBC correlations from H-5 (*δ*_H_ 6.54) to C-4 (*δ*_C_ 69.6) and C-9 (*δ*_C_ 113.3). A cross-peak between the methoxy protons (*δ*_H_ 3.68) and C-8 (*δ*_C_ 155.1) fixed the O-methyl group at C-8. The isochromane connectivity was supported by correlations from H_2_-1 (*δ*_H_ 4.43 and 4.55) to C-3 (*δ*_C_ 74.8), C-4 (*δ*_C_ 69.6), and C-9 (*δ*_C_ 113.3), as well as from H-3 (*δ*_H_ 3.31) to C-1 (*δ*_C_ 63.6). On this basis, the constitution of **2** was determined as 8-methoxy-3-methylisochromane-4,6-diol, the C-5-demethylated counterpart of **7**.

**Figure 3:**
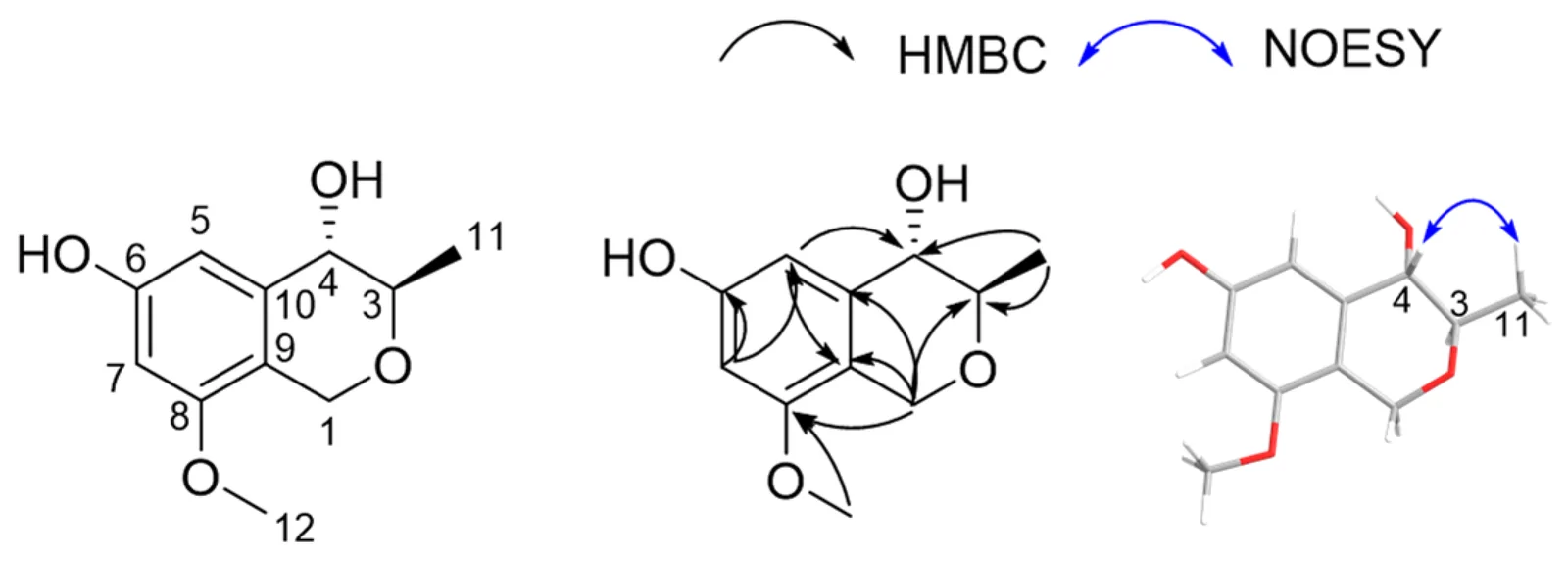
Structure of compound **2** with selected long-range HMBC and through-space NOESY correlations.

A NOESY cross-peak between H-4 (*δ*_H_ 4.00) and CH_3_-11 (*δ*_H_ 1.24), together with enhancement of the methyl resonance upon selective irradiation of H-4, indicated that these groups were cofacial. The coupling constant between H-3 and H-4 (*J* = 6.4 Hz) was consistent with a *trans*-diaxial relationship [21]. Biosynthetic considerations and comparison with the co-isolated analogue **7** supported a tentative 3*R*,4*S* assignment. Because neither single-crystal X-ray diffraction nor ECD calculations were available, the absolute configuration cannot be considered definitive. Accordingly, compound **2** was tentatively assigned as (3*R*,4*S*)-8-methoxy-3-methylisochromane-4,6-diol (Figure 3).

The known metabolites were assigned as 2(1H)-quinolinone (**3**), 4-hydroxy-3-methoxy-2(1H)-quinolinone (**4**), penicinoline E (**5**), methyl marinamide (**6**), (3*S*,4*R*)-6-hydroxy-8-methoxy-3,5-dimethylisochromanol (**7**), methyl 3-indoleacetate (**8**), 1H-indole-3-carboxamide (**9**), and methyl 1H-indole-3-carboxylate (**10**) by matching their NMR data with published values [18,19,20,21,22,23,24,25,26,27,28,29].

### 2.2. Inhibition of NO Production and In Silico Target Analysis

**Figure 4:**
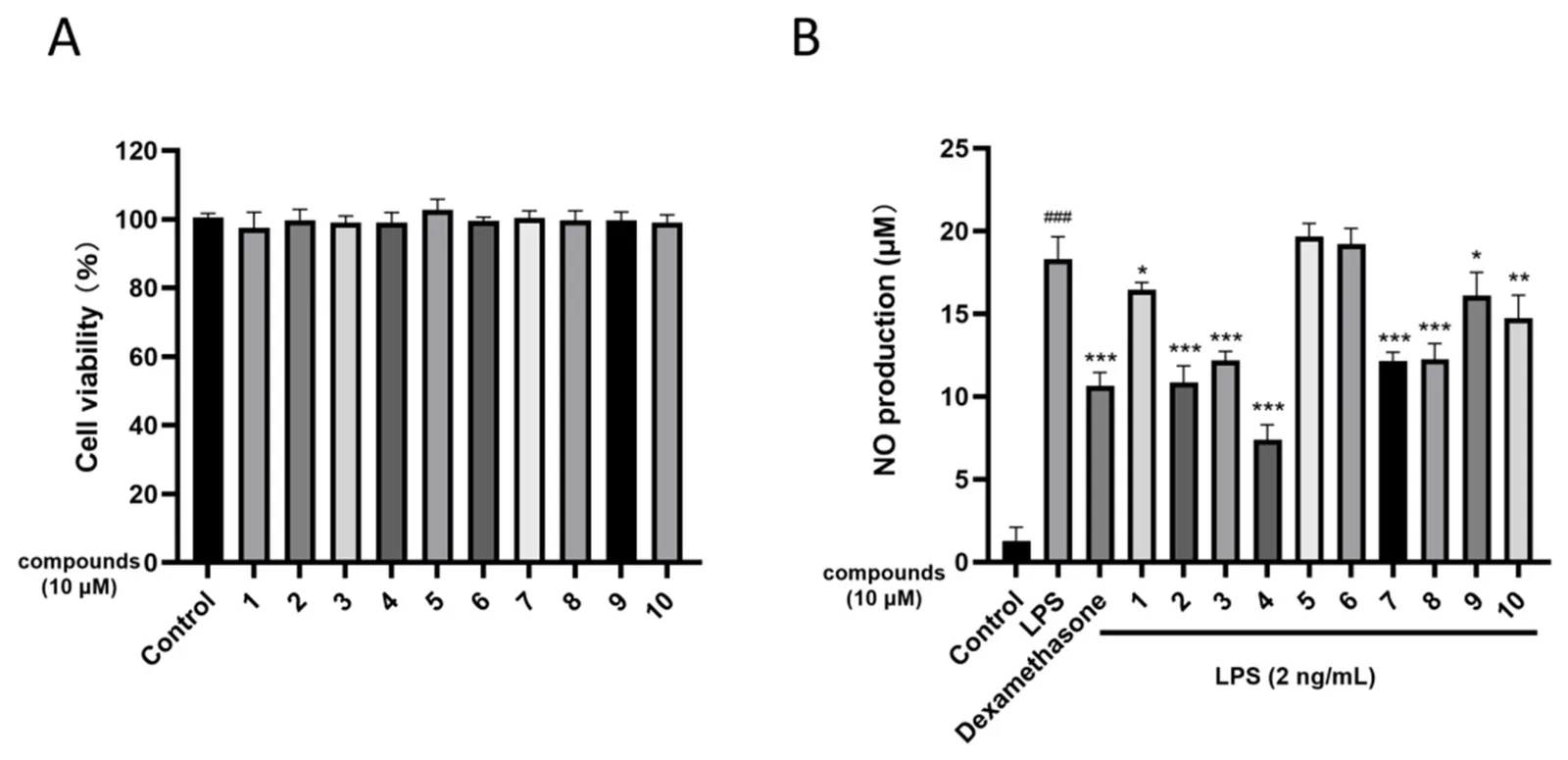
Effects of compounds **1**–**10** at 10 μM on RAW 264.7 macrophages. (**A**) Cell viability after 24 h of treatment (*n* = 3). (**B**) NO accumulation in LPS-stimulated cells treated with the indicated compounds (*n* = 6). Data are presented as the mean ± SEM. ^###^ *p* < 0.001 versus the untreated control; * *p* < 0.05, ** *p* < 0.01, and *** *p* < 0.001 versus the LPS-treated model group.

Because excessive NO release from activated macrophages is widely used as an indicator of inflammatory activation, the isolates were evaluated in LPS-stimulated RAW 264.7 cells. The MTT assay showed that none of compounds **1**–**10** reduced cell viability after 24 h at 10 μM (Figure 4A), supporting the use of this concentration in the initial activity screen. At 10 μM, compounds **1**–**4** and **7**–**10** significantly decreased LPS-induced NO accumulation, whereas compounds **5** and **6** showed little or no inhibitory activity (Figure 4B). Dose–response analysis of the active compounds yielded IC_50_ values ranging from 18.67 to 40.05 μM (Table 3). Of the two newly characterized metabolites, compound **2** was more active than compound **1**, with IC_50_ values of 19.01 and 40.05 μM, respectively. Because these findings are based solely on NO production, additional inflammatory markers, including iNOS, TNF-α, and IL-6, should be examined before broader anti-inflammatory activity is inferred.

**Figure 5:**
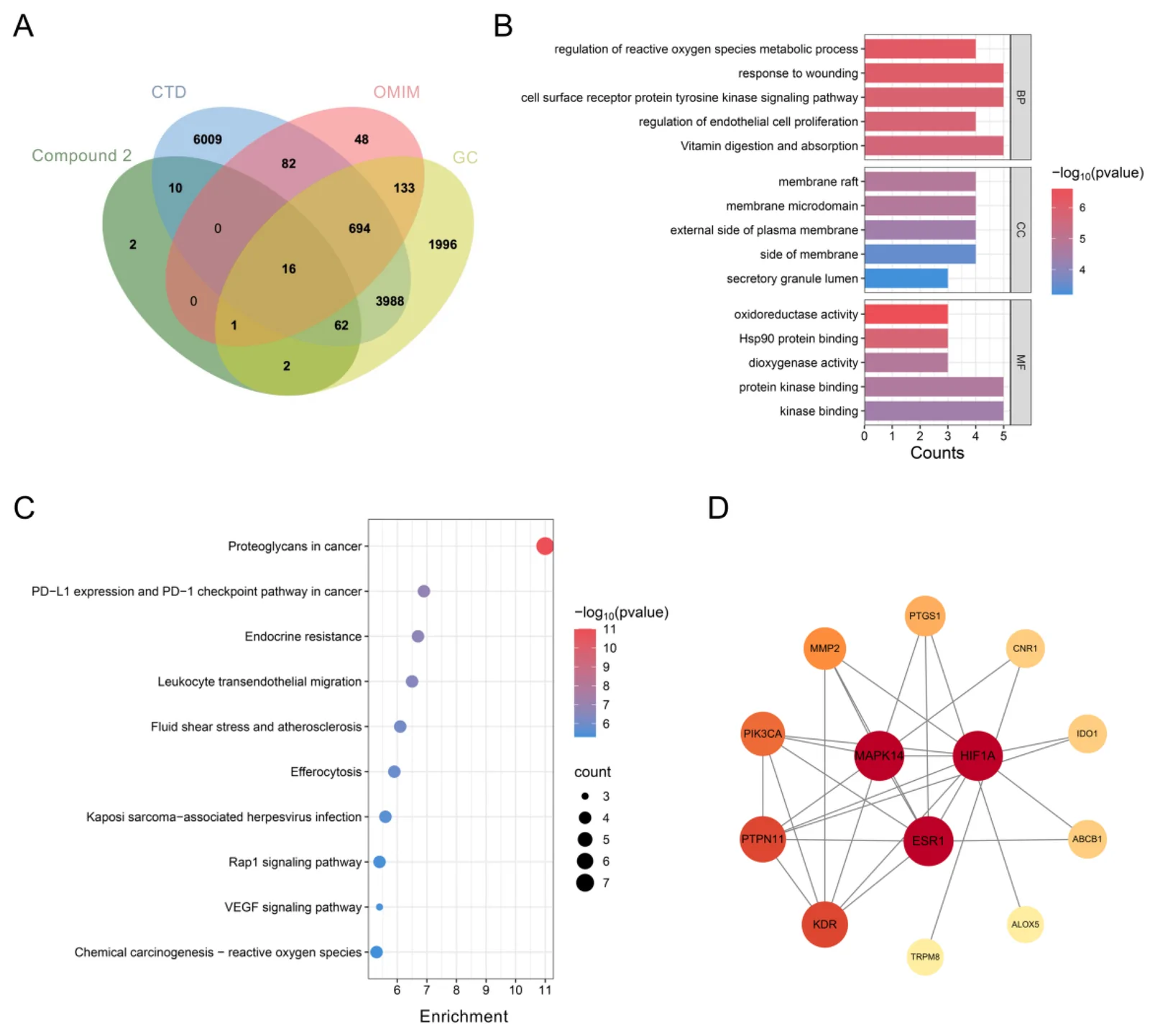
Computational prioritization of inflammation-associated targets for compound **2**. (**A**) Intersection of predicted compound targets with gene sets retrieved from three inflammation databases. (**B**) Leading GO terms in the BP, CC, and MF categories. (**C**) Ten highest-ranked KEGG pathways displayed as a bubble plot. (**D**) PPI network constructed from the 16 shared targets.

Compound **2**, the more active of the two new metabolites in the NO assay, was selected for an exploratory network-pharmacology analysis. SwissTargetPrediction returned 81 putative protein targets. Searches of the CTD, OMIM, and GeneCards resources yielded 10,861, 974, and 6892 inflammation-associated genes, respectively. Intersection of these four sets produced 16 candidates: *ALOX5*, *KDR*, *TRPM8*, *PIK3CA*, *HIF1A*, *MMP2*, *ABCB1*, *MAPT*, *PTGS1*, *TTR*, *ESR1*, *CCR3*, *IDO1*, *MAPK14*, *CNR1*, and *PTPN11* (Figure 5A). GO analysis linked the shared set mainly to regulation of reactive oxygen species metabolism, wound responses, receptor tyrosine kinase signaling, endothelial-cell proliferation, membrane microdomains, and oxidoreductase or kinase-binding functions (Figure 5B). The KEGG output included leukocyte transendothelial migration, efferocytosis, fluid shear stress and atherosclerosis, VEGF signaling, Rap1 signaling, and chemical carcinogenesis–reactive oxygen species (Figure 5C). These associations suggest possible connections to vascular permeability, cell movement, and oxidative stress, but they should be interpreted as predictions rather than demonstrated mechanisms. Within the PPI network, *MAPK14*, *HIF1A*, and *ESR1* were the principal hub targets (Figure 5D) and were therefore taken forward for docking.

**Figure 6:**
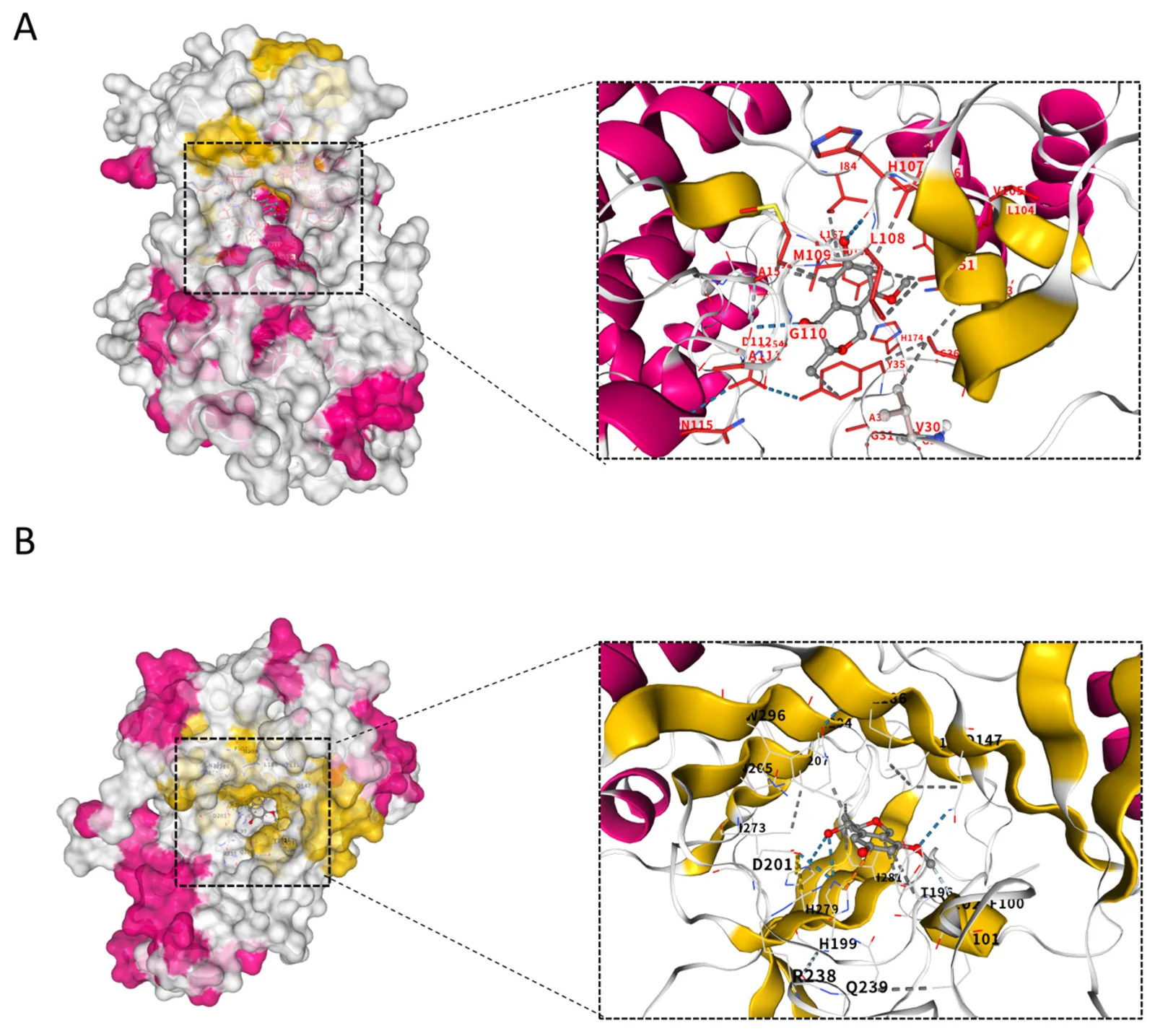

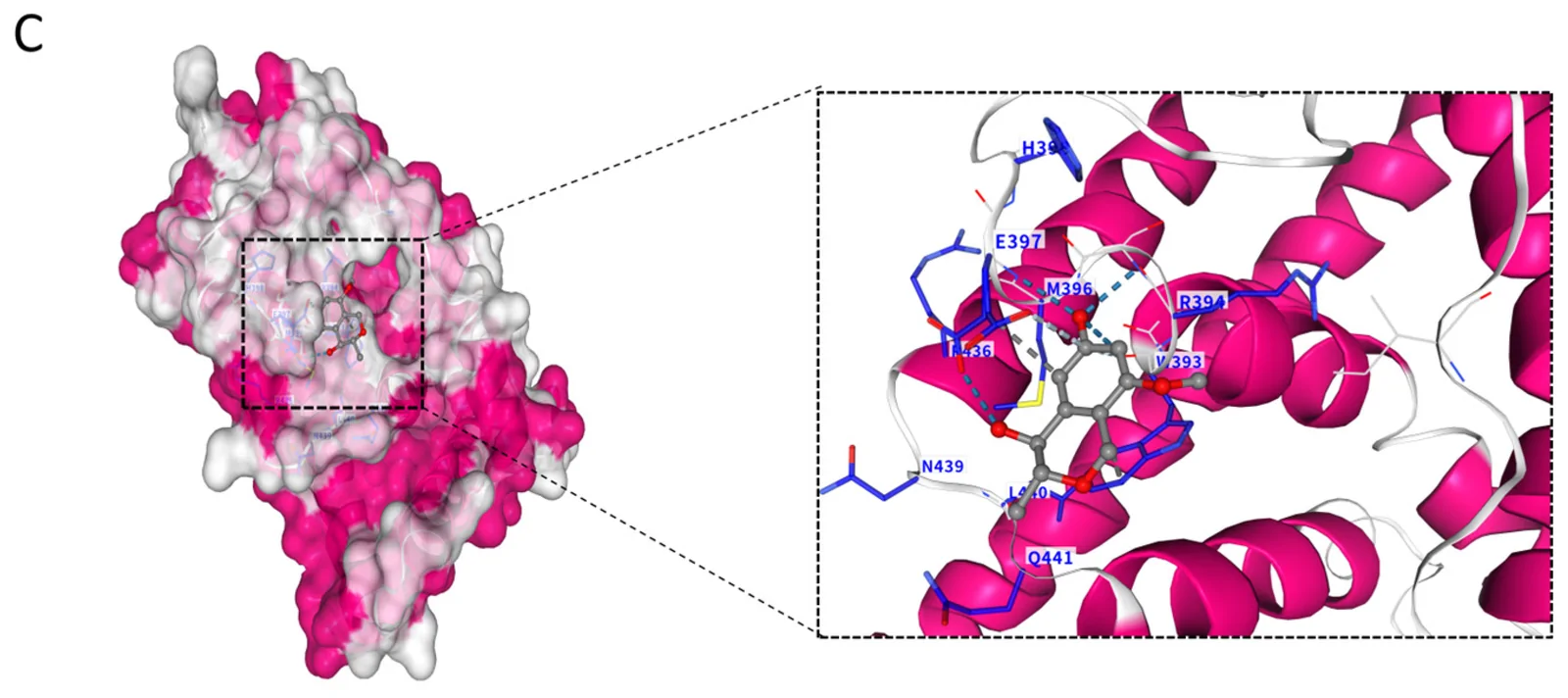
Docking models reported for compound **2** with the three PPI-network hub targets: (**A**) MAPK14, (**B**) HIF1A, and (**C**) ESR1. Residues predicted to interact with the ligand are indicated.

Docking was performed to assess whether compound **2** could adopt plausible binding poses within the selected protein structures. In the MAPK14 model, the ligand formed predicted hydrogen bonds with H107, L108, D112, and N115 and yielded a Vina score of −7.4 kcal/mol (Figure 6A). The model labeled HIF1A showed polar contacts with D201, H199, R238, and Q239 and hydrophobic contacts with V196, I195, and V273, with a score of −6.6 kcal/mol (Figure 6B). In the model labeled ESR1, E397, M396, R394, N439, and Q441 were predicted to participate in hydrogen bonding, whereas V393 and V409 contributed hydrophobic contacts; the corresponding score was −4.7 kcal/mol (Figure 6C). Of the three models, the MAPK14 complex produced the most favorable score. These in silico results identify interactions for experimental investigation but do not demonstrate direct target engagement.

## 3. Materials and Methods

### 3.1. General Experimental Procedures

Optical rotation data were obtained with an Autopol IV polarimeter (Rudolph Research Analytical, Hackettstown, NJ, USA). ECD measurements were performed on a Jasco J-810-150S spectropolarimeter (Jasco, Tokyo, Japan) from 200 to 400 nm. HRESIMS analyses were conducted using an AB Sciex TripleTOF 4600 mass spectrometer (AB Sciex, Framingham, MA, USA). One- and two-dimensional NMR spectra, including ^1^H, ^13^C, HSQC, HMBC, and NOESY experiments, were acquired on a Bruker Avance II 500 MHz instrument (Bruker BioSpin, Rheinstetten, Germany) in DMSO-d_6_, with the residual solvent resonance used for chemical shift referencing. Silica gel (100–200 and 200–300 mesh; Qingdao Ocean Chemical Co., Ltd., Qingdao, China) and Sephadex LH-20 (Cytiva, Uppsala, Sweden) were used for column chromatographic separations. Analytical TLC was carried out on silica gel GF_254_ precoated plates (Qingdao Ocean Chemical Co., Ltd.), with chromatographic zones detected under UV illumination at 254 and 365 nm. Semipreparative separations were performed on Agilent 1260 (Santa Clara, CA, USA) and Shimadzu LC-20AR HPLC (Shimadzu Corporation, Kyoto, Japan) systems fitted with an XB-C18 column (10 × 250 mm, 5 μm) and monitored at 210 and 254 nm. Analytical-grade solvents were used for extraction and routine chromatographic procedures, while HPLC-grade solvents were employed for HPLC purification. Ultrapure water was used in all experiments. RAW 264.7 mouse macrophages were supplied by Procell (Wuhan, China). High-glucose DMEM, penicillin–streptomycin, and fetal bovine serum were sourced from Gibco (Grand Island, NY, USA), Solaris (Beijing, China), and ExCell Bio (Shanghai, China), respectively. The nitric oxide detection kit was purchased from Beyotime Biotechnology (Shanghai, China), and MTT was obtained from Solarbio (Beijing, China).

### 3.2. Fungal Material

The endophytic fungus HJT-A-10 was derived from root tissues of *Rhodiola tibetica*, which was collected at an altitude of 4500 m in Langkazi County (Shannan, Tibet, China; 28°56′41″ N, 90°20′10″ E). Repeated subculturing of single colonies on potato dextrose agar (PDA) afforded a pure isolate. Taxonomic identification was based on morphological features together with ITS sequence analysis. The ITS1–5.8S–ITS2 rDNA region was amplified and sequenced, and the sequence was deposited in GenBank under accession number PX108325. Comparison with related sequences using BLAST+ 2.13.0, followed by neighbor-joining phylogenetic analysis in MEGA 11, supported assignment of the strain to *Penicillium steckii*. A voucher culture was deposited at the China General Microbiological Culture Collection Center (CGMCC 40167), and a working culture is preserved at the College of Life and Health, Dalian University, under the strain number HJT-A-10.

### 3.3. Fermentation, Extraction and Isolation of Compounds

*Penicillium steckii* HJT-A-10 was grown in PDB at 28 °C and 200 rpm for 2 days to establish the seed culture, which was then introduced into sterile rice medium and incubated statically at 28 °C for 30 days. The resulting fermented mass was ultrasonically extracted three times with 75% aqueous MeOH, and the combined solutions were evaporated under reduced pressure to give 528.5 g of total crude extract. This residue was suspended in H_2_O and extracted with EtOAc to obtain an EtOAc-soluble fraction (90.7 g).

This fraction was applied to silica gel CC and eluted with gradients of petroleum ether–EtOAc (100:0 → 1:1) and CH_2_Cl_2_–MeOH (100:0 → 0:100). Fractions with matching TLC patterns were pooled into 15 fractions (Fr.1–15). Fr.6 was re-subjected to silica gel CC eluted with petroleum ether–EtOAc (100:0 → 0:100), producing 17 subfractions. Fr.6-1 was separated on Sephadex LH-20 (CH_2_Cl_2_/MeOH, 1:1), and the second subfraction obtained was purified by semipreparative HPLC (MeOH/H_2_O, 42:58) to afford **2** (1.7 mg, tR = 20 min) and **3** (1.9 mg, tR = 26 min). Fr.6-2 was sequentially purified by Sephadex LH-20 and semipreparative HPLC (MeOH/H_2_O, 15:85) to yield **6** (1.9 mg, tR = 13 min) and **7** (2.9 mg, tR = 20 min). Semipreparative HPLC of Fr.6-3 with MeOH/H_2_O (48:52) gave **4** (1.3 mg, tR = 27 min), **1** (1.6 mg, tR = 32 min), and **5** (1.6 mg, tR = 36 min). Fr.6-4, after Sephadex LH-20 and semipreparative HPLC (MeOH/H_2_O, 20:80), gave **10** (3.0 mg, tR = 20 min). Fr.6-5 was treated on Sephadex LH-20 and then purified by prep-TLC (petroleum ether–EtOAc, 1:1) to yield **8** (2.3 mg) and **9** (3.6 mg).

### 3.4. Spectroscopic Data

4,7-Dihydroxy-3-methoxyquinolin-2(1H)-one (**1**): purple powder; ${\left[\alpha \right]}_{D}^{25}$ − 14.0 (c 0.14, MeOH); ^1^H and ^13^C NMR data, see Table 1; HRESIMS *m*/*z* 230.0429 [M + Na]^+^ (calcd for C_10_H_9_NO_4_Na, 230.0424).

(3*R*,4*S*)-8-methoxy-3-methylisochromane-4,6-diol (**2**): yellow oil; ${\left[\alpha \right]}_{D}^{20}$ + 50.0 (c 0.08, MeOH); ^1^H and ^13^C NMR data, see Table 2; HRESIMS *m*/*z* 233.0788 [M + Na]^+^ (calcd for C_11_H_14_O_4_Na, 233.0784).

### 3.5. Cell Culture

RAW 264.7 murine macrophages (Procell) were cultured in high-glucose DMEM supplemented with 10% FBS, 100 U/mL penicillin, and 100 μg/mL streptomycin, at 37 °C in a humidified 5% CO_2_ incubator. Logarithmically growing cells were used throughout this study.

### 3.6. Anti-Inflammatory Test

RAW 264.7 macrophages, seeded in 96-well plates at about 60% confluence, were treated for 24 h with LPS (2 ng/mL), both in the absence and presence of the test substances. All compounds were subjected to HPLC purity verification, which yielded values > 95%. Dexamethasone functioned as the positive control. Viability of cells was evaluated by the MTT assay according to an earlier description [30]. As an indicator of NO generation, nitrite in the supernatant was quantified with a kit from Beyotime (Shanghai, China), following the accompanying instructions.

### 3.7. Network Pharmacological Analysis

The canonical SMILES string of compound **2** was submitted to SwissTargetPrediction to obtain putative protein targets. Genes associated with inflammation were retrieved independently from the CTD, OMIM, and GeneCards databases. Genes shared by the predicted compound-target set and the three inflammation-related datasets were retained as candidate targets. GO and KEGG enrichment analyses were performed using Metascape (https://metascape.org/gp/) (accessed on 25 June 2026). Enriched terms were ranked by *p* value; the five highest-ranking terms in each GO category (BP, CC, and MF) and the ten highest-ranking KEGG pathways were selected for visualization [31,32].

### 3.8. Molecular Docking

Molecular docking was performed using the CB-Dock web server (https://cadd.labshare.cn/cb-dock; accessed on 16 July 2026). The structures specified for the three candidate targets were MAPK14 (PDB ID: 2LGC), HIF1A (PDB ID: 1H2K), and ESR1 (PDB ID: 2GFA). Compound **2** was submitted as a SMILES string, and its three-dimensional conformations were generated automatically by the server. Blind docking was conducted over the entire protein surface using the integrated AutoDock Vina 1.2.0 scoring function with default parameters and a flexible ligand. For each protein, the pose with the lowest Vina score was selected for interaction analysis [33].

### 3.9. Statistical Analysis

Biological replicates numbered three for the MTT assay and six for the NO assay; all samples were run in triplicate for technical repeats. Results are given as means ± SEM. Intergroup differences were examined via one-way ANOVA with Dunnett’s correction, and significance was accepted at *p* < 0.05. Analyses were executed with GraphPad Prism 5.0, and IC_50_ determination relied on nonlinear regression fitted to a four-parameter logistic function [34].

## 4. Conclusions

Chemical investigation of *Penicillium steckii* HJT-A-10 yielded two previously undescribed metabolites, 4,7-dihydroxy-3-methoxyquinolin-2(1H)-one (**1**) and (3*R*,4*S*)-8-methoxy-3-methylisochromane-4,6-diol (**2**), together with eight known compounds; the absolute configuration of **2** remains tentative. In LPS-stimulated RAW 264.7 macrophages, compounds **1**–**4** and **7**–**10** reduced NO accumulation, with IC_50_ values of 18.67–40.05 μM. Compound **2** was the more active of the two new metabolites (IC_50_ = 19.01 μM). Network analysis prioritized MAPK14, HIF1A, and ESR1, while the MAPK14 docking model yielded the most favorable Vina score. Because the computational analyses are hypothesis-generating, direct target-engagement assays, evaluation of additional inflammatory mediators, and in vivo studies are required before the pharmacological significance of compound **2** can be established.

## Figures and Tables

**Table 1:** NMR spectroscopic assignments for compound **1** in DMSO-d_6_ (^1^H, 500 MHz; ^13^C, 125 MHz).

Position	1	
	*δ*_C_	*δ*_H_ (Multiplicity, *J* in Hz)
1-NH		10.09, s, 1H
2	160.2	
3	128.4	
4	152.2	
4-OH		11.15, s, 1H
5	123.9	7.56, d (8.5), 1H
6	111.0	6.61, dd (8.5, 2.5), 1H
7	158.7	
8	99.6	6.63, d (2.5), 1H
9	137.4	
10	108.5	
11	59.3	3.69, s, 3H

**Table 2:** NMR spectroscopic assignments for compound **2** in DMSO-d_6_ (^1^H, 500 MHz; ^13^C, 125 MHz).

Position	2	
	*δ*_C_	*δ*_H_ (Multiplicity, *J* in Hz)
1	63.6	Ha 4.43, dd (15.0, 1.5), 1H Hb 4.55, d (15.0), 1H
3	74.8	3.31, q (6.2), 1H
4	69.6	4.00, t (6.4), 1H
5	104.7	6.54, d (3.0), 1H
6	157.0	
7	97.0	6.24, d (2.0), 1H
8	155.1	
9	113.3	
10	140.1	
11	18.6	1.24, d (6.0), 3H
12	55.1	3.68, s, 3H

**Table 3:** Effects of compounds **1**–**4** and **7**–**10** on RAW 264.7 cell viability and LPS-induced NO accumulation.

Compound	Cell Viability at 10 μM (%)	NO Inhibition at 10 μM (%)	NO IC_50_ (μM)
**1**	97 ± 3	11 ± 3	40.05
**2**	100 ± 2	44 ± 4	19.01
**3**	99 ± 1	36 ± 3	27.73
**4**	99 ± 2	64 ± 3	18.67
**7**	100 ± 1	36 ± 2	23.61
**8**	100 ± 2	35 ± 4	35.42
**9**	100 ± 1	13 ± 4	30.45
**10**	99± 1	21 ± 5	32.14

## Data Availability

The data presented in this study are available in this article and Supplementary Materials.

## Supplementary Materials

The following supporting information can be downloaded at: https://www.mdpi.com/article/10.3390/molecules31173109/s1, Figure S1: The phylogenetic tree of fungal strain HTJ-A-10 based on ITS gene sequences; Figure S2: HRESIMS spectrum of compound **1**; Figure S3: ^1^H NMR spectrum of compound **1** in DMSO-*d*_6_ (500 MHz); Figure S4: ^13^C NMR spectrum of compound **1** in DMSO-*d*_6_ (125 MHz); Figure S5: HSQC spectrum of compound **1** in DMSO-*d*_6_; Figure S6: HMBC spectrum of compound **1** in DMSO-*d*_6_; Figure S7: HRESIMS spectrum of compound **2**; Figure S8: ^1^H NMR spectrum of compound **2** in DMSO-*d*_6_ (500 MHz); Figure S9: ^13^C NMR spectrum of compound **2** in DMSO-*d*_6_ (125 MHz); Figure S10: HSQC spectrum of compound **2** in DMSO-*d*_6_; Figure S11: HMBC spectrum of compound **2** in DMSO-*d*_6_; Figure S12: COSY spectrum of compound **2** in DMSO-*d*_6_; Figure S13: NOESY spectrum of compound **2** in DMSO-*d*_6_; Figure S14: ^1^H NMR spectrum of compound **3** in DMSO-*d*_6_ (500 MHz); Figure S15: ^1^H NMR spectrum of compound **4** in DMSO-*d*_6_ (500 MHz); Figure S16: ^1^H NMR spectrum of compound **5** in DMSO-*d*_6_ (500 MHz); Figure S17: ^1^H NMR spectrum of compound **6** in DMSO-*d*_6_ (500 MHz); Figure S18: ^1^H NMR spectrum of compound **7** in DMSO-*d*_6_ (500 MHz); Figure S19: ^1^H NMR spectrum of compound **8** in DMSO-*d*_6_ (500 MHz); Figure S20: ^1^H NMR spectrum of compound **9** in DMSO-*d*_6_ (500 MHz); Figure S21: ^1^H NMR spectrum of compound **10** in DMSO-*d*_6_ (500 MHz).
