# Supplementary material for: Chemical Constituents of the Endophytic Fungus Penicillium steckii HJT-A-10 from Rhodiola tibetica and Their Anti-Inflammatory Activities
Source: Molecules. 2026 Sep 4;31(17):3109. doi: 10.3390/molecules31173109 (PMC13567667; doi:10.3390/molecules31173109)
Supplement: Supplementary file 1 [file molecules-31-03109-s001.zip › molecules-4486661-supplementary.pdf]

Supporting information

Chemical Constituents of the Endophytic Fungus *Penicillium steckii* HJT-A-10 from *Rhodiola tibetica* and Their Anti-Inflammatory Activities

Yujie Lu, Haoyu Xiang, Yanjie Xu, Xinge Yu, Janar Jenis, Baomin Feng, Dongliang Xiao and Xuan Lu

Table of Contents

Figure S1 The phylogenetic tree of fungal strain HTJ-A-10 based on ITS gene sequences.

Figure S2 HRESIMS spectrum of compound **1**.

Figure S3 ^{1}H NMR spectrum of compound **1** in DMSO-d_6 (500 MHz)

Figure S4 ^{13}C NMR spectrum of compound **1** in DMSO-d_6(125 MHz)

Figure S5 HSQC spectrum of compound **1** in DMSO-d_6

Figure S6 HMBC spectrum of compound **1** in DMSO-d_6

Figure S7 HRESIMS spectrum of compound **2**.

Figure S8 ^{1}H NMR spectrum of compound **2** in DMSO-d_6 (500 MHz)

Figure S9 ^{13}C NMR spectrum of compound **2** in DMSO-d_6 (125 MHz)

Figure S10 HSQC spectrum of compound **2** in DMSO-d_6

Figure S11 HMBC spectrum of compound **2** in DMSO-d_6

Figure S12 COSY spectrum of compound **2** in DMSO-d_6

Figure S13 NOESY spectrum of compound **2** in DMSO-d_6

Figure S14 ^{1}H NMR spectrum of compound **3** in DMSO-d_6 (500 MHz)

Figure S15 ^{1}H NMR spectrum of compound **4** in DMSO-d_6 (500 MHz)

Figure S16 ^{1}H NMR spectrum of compound **5** in DMSO-d_6 (500 MHz)

Figure S17 ^{1}H NMR spectrum of compound **6** in DMSO-d_6 (500 MHz)

Figure S18 ^{1}H NMR spectrum of compound **7** in DMSO-d_6 (500 MHz)

Figure S19 ^{1}H NMR spectrum of compound **8** in DMSO-d_6 (500 MHz)

Figure S20 ^{1}H NMR spectrum of compound **9** in DMSO-d_6 (500 MHz)

Figure S21 ^{1}H NMR spectrum of compound **10** in DMSO-d_6 (500 MHz)

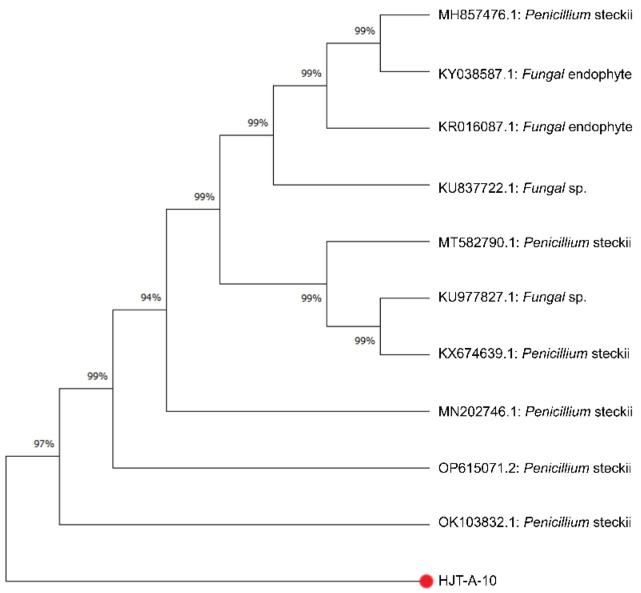

Figure S1. The phylogenetic tree of fungal strain HTJ-A-10 based on ITS gene sequences.

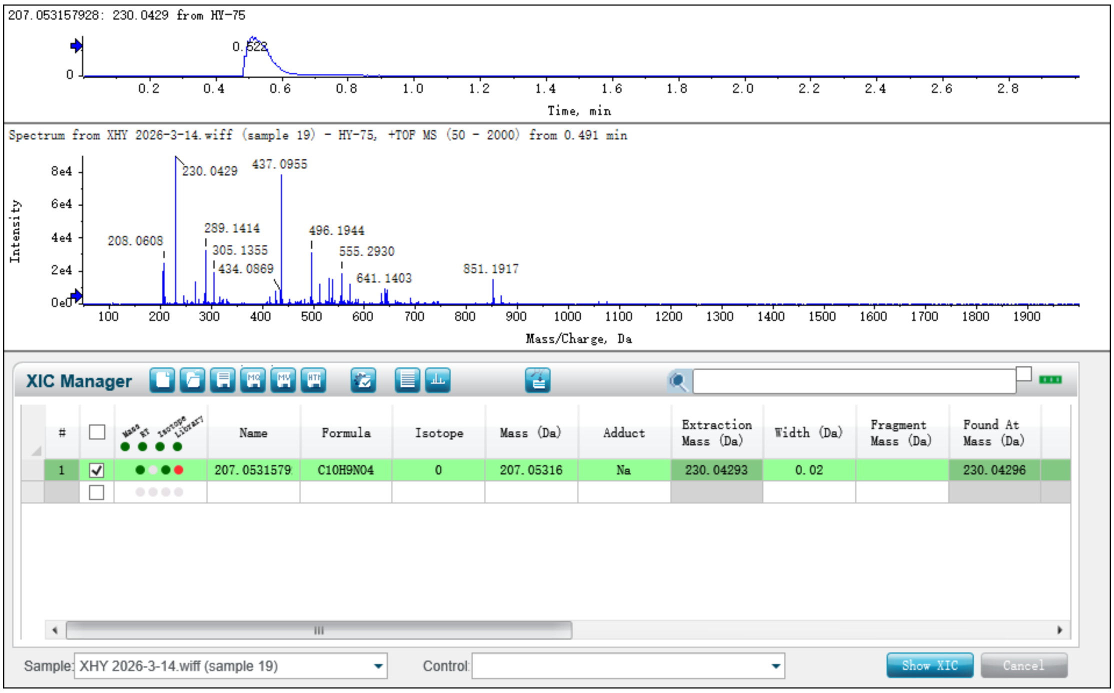

#		Name	Formula	Isotope	Mass (Da)	Adduct	Extraction Mass (Da)	Width (Da)	Fragment Mass (Da)	Found At Mass (Da)
1	☑	207.0531579	C10H9NO4	0	207.05316	Na	230.04293	0.02		230.04296

Figure S2. HRESIMS spectrum of compound **1**.

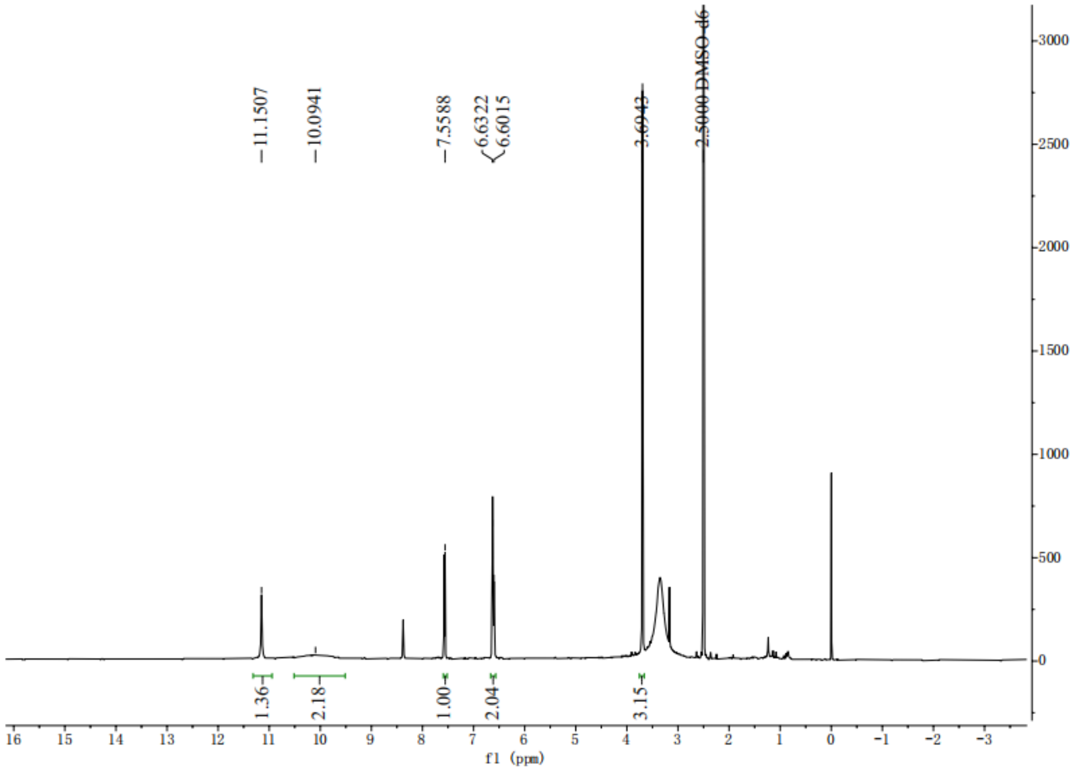

Figure S3. ^{1}H NMR spectrum of compound **1** in DMSO-d_6 (500 MHz).

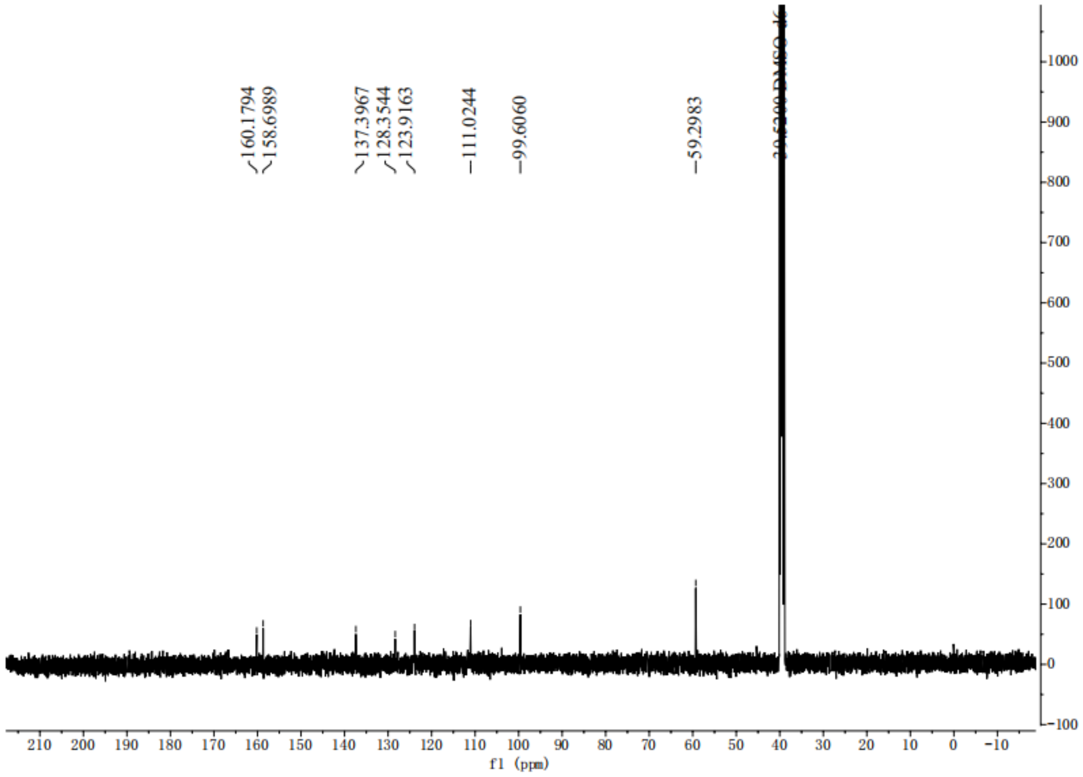

Figure S4. ^{13}C NMR spectrum of compound **1** in DMSO-d_6 (125 MHz).

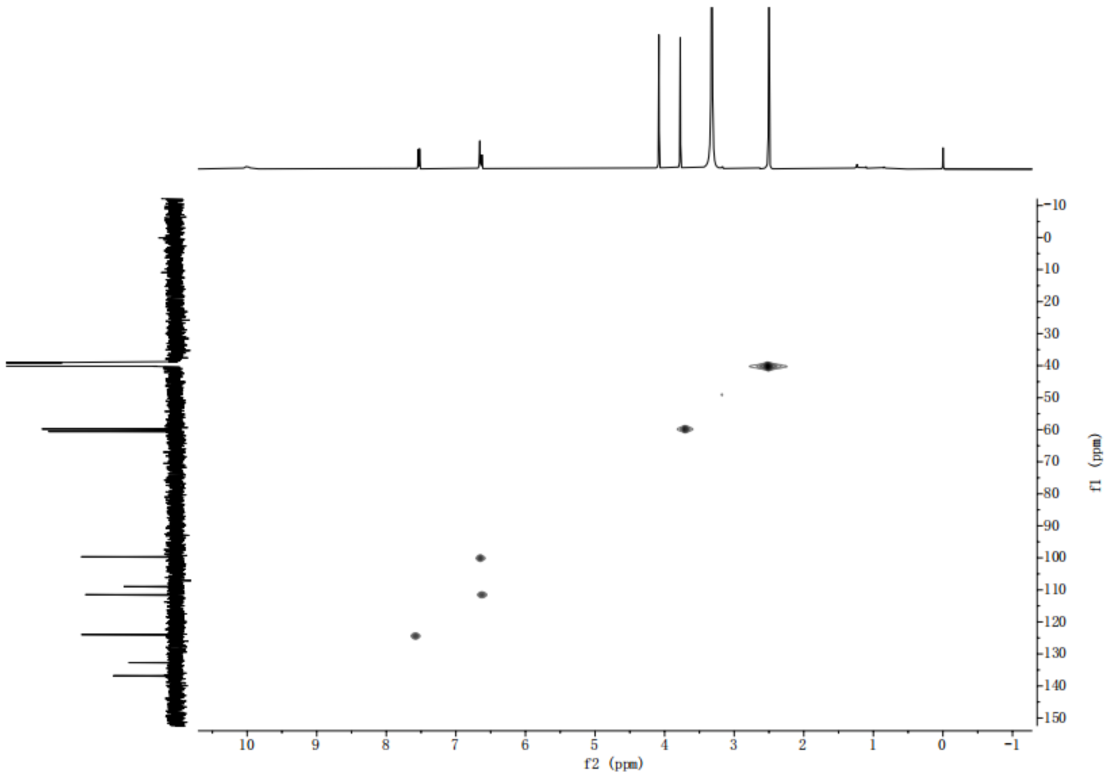

Figure S5. HSQC spectrum of compound **1** in DMSO-d_6.

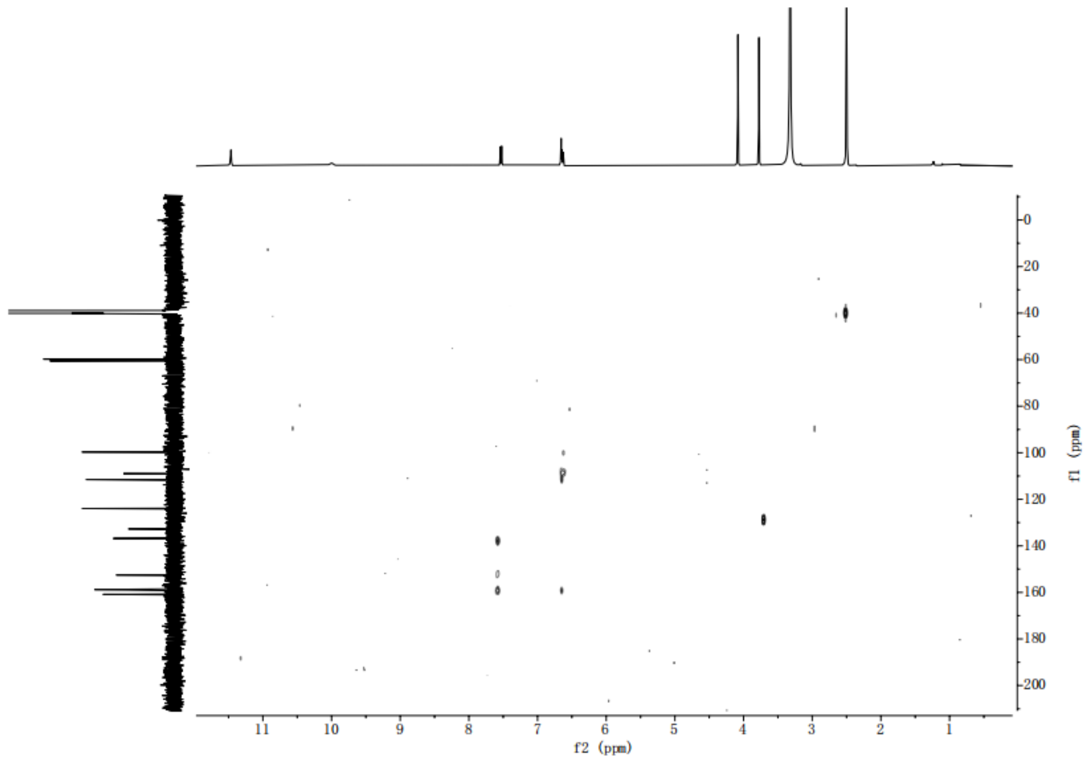

Figure S6. HMBC spectrum of compound **1** in DMSO-d_6.

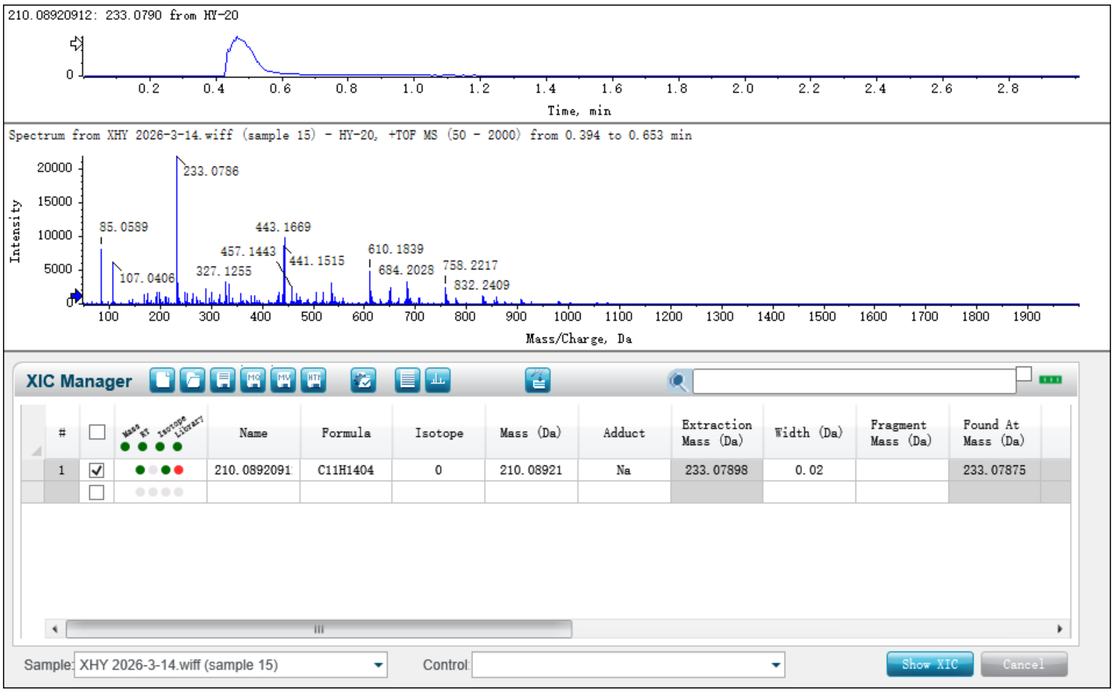

Figure S7. HRESIMS spectrum of compound **2**.

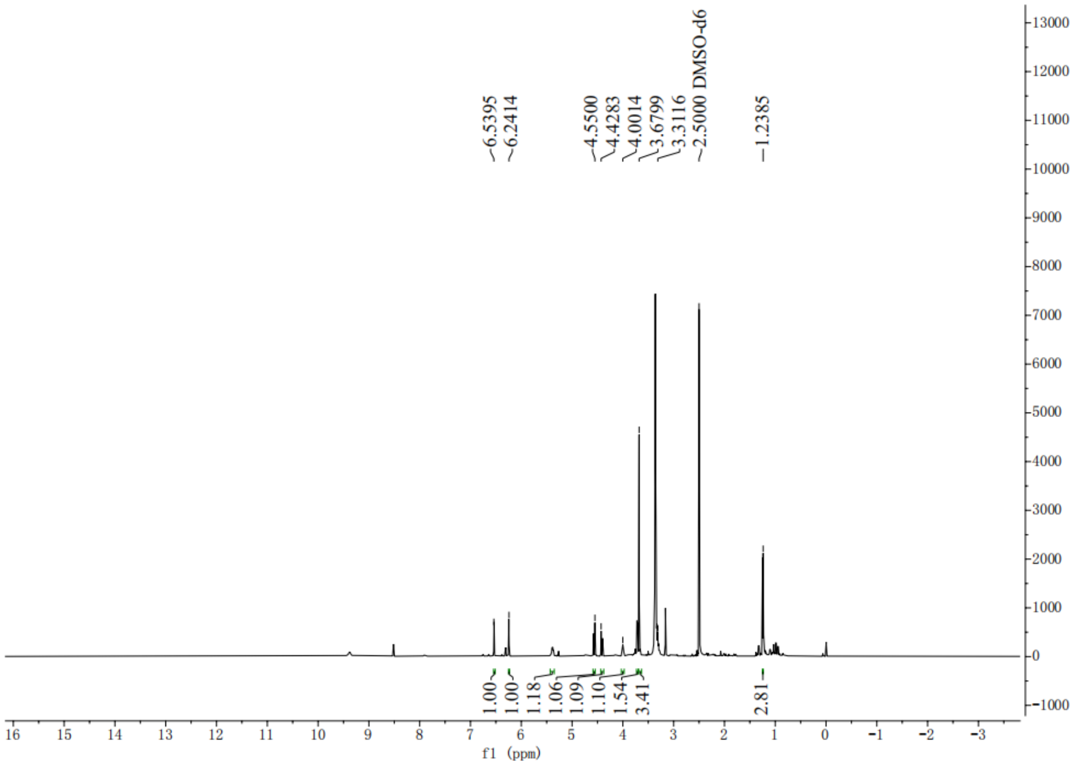

Figure S8. ^{1}H NMR spectrum of compound **2** in DMSO-d_6 (500 MHz).

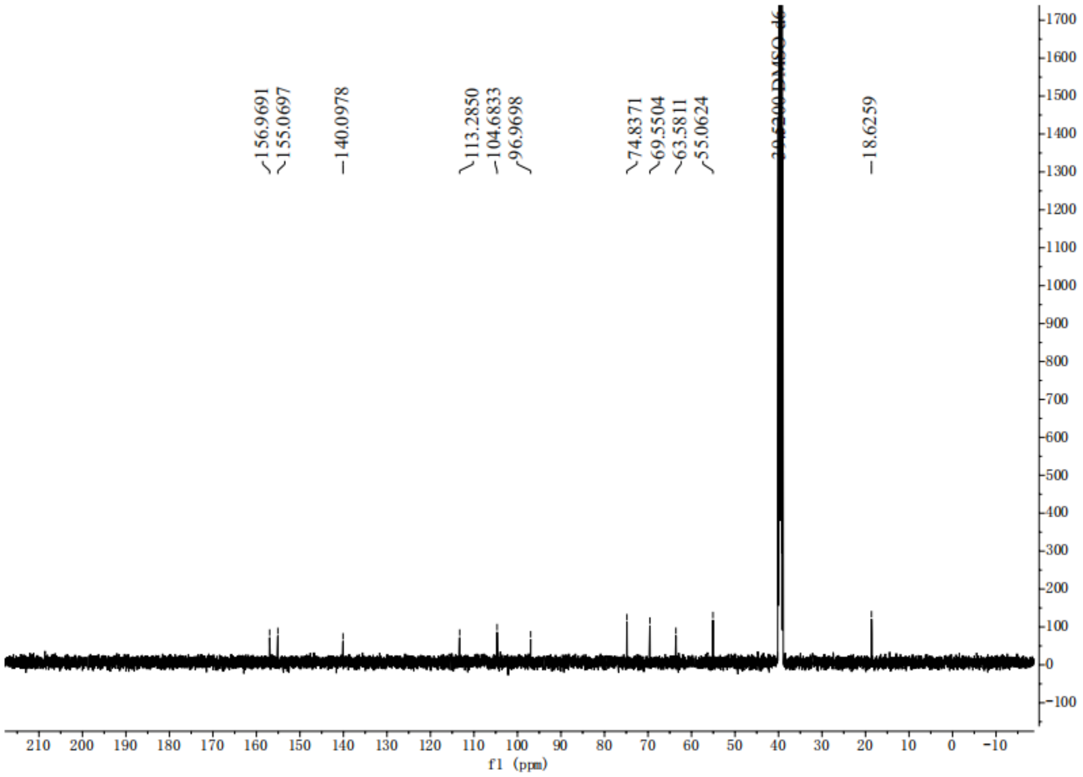

Figure S9. ^{13}C NMR spectrum of compound **2** in DMSO-d_6 (125 MHz).

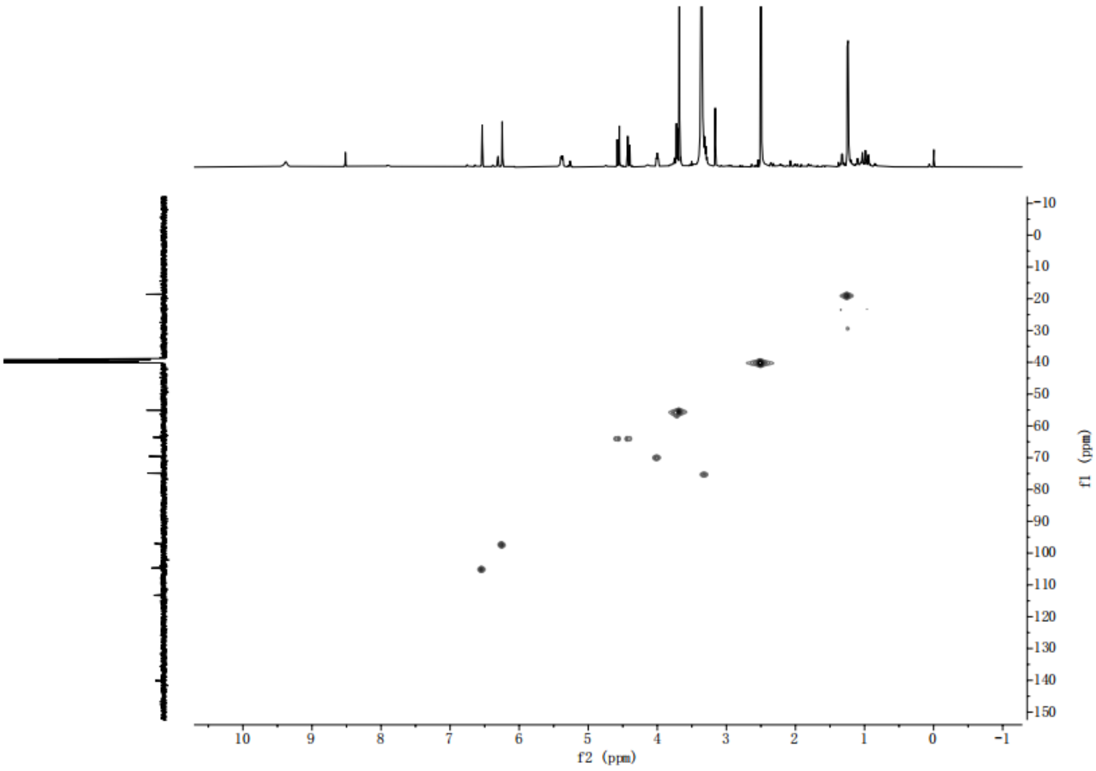

Figure S10. HSQC spectrum of compound **2** in DMSO-d_6.

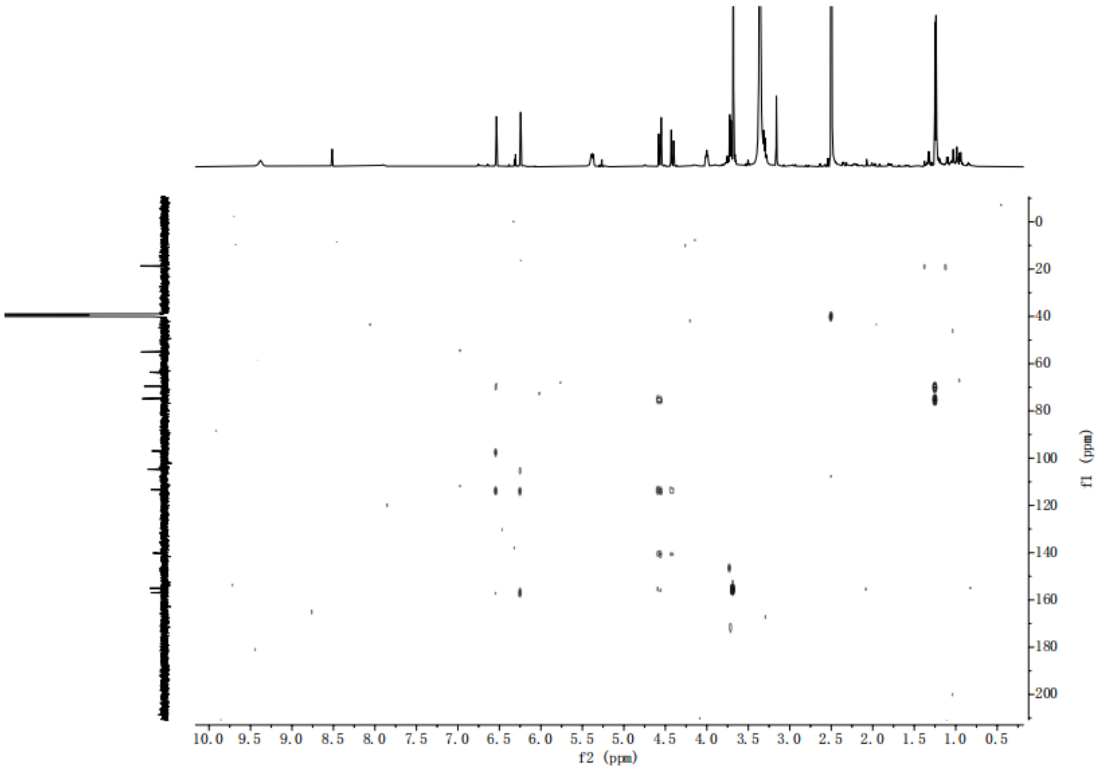

Figure S11. HMBC spectrum of compound **2** in DMSO-d_6.

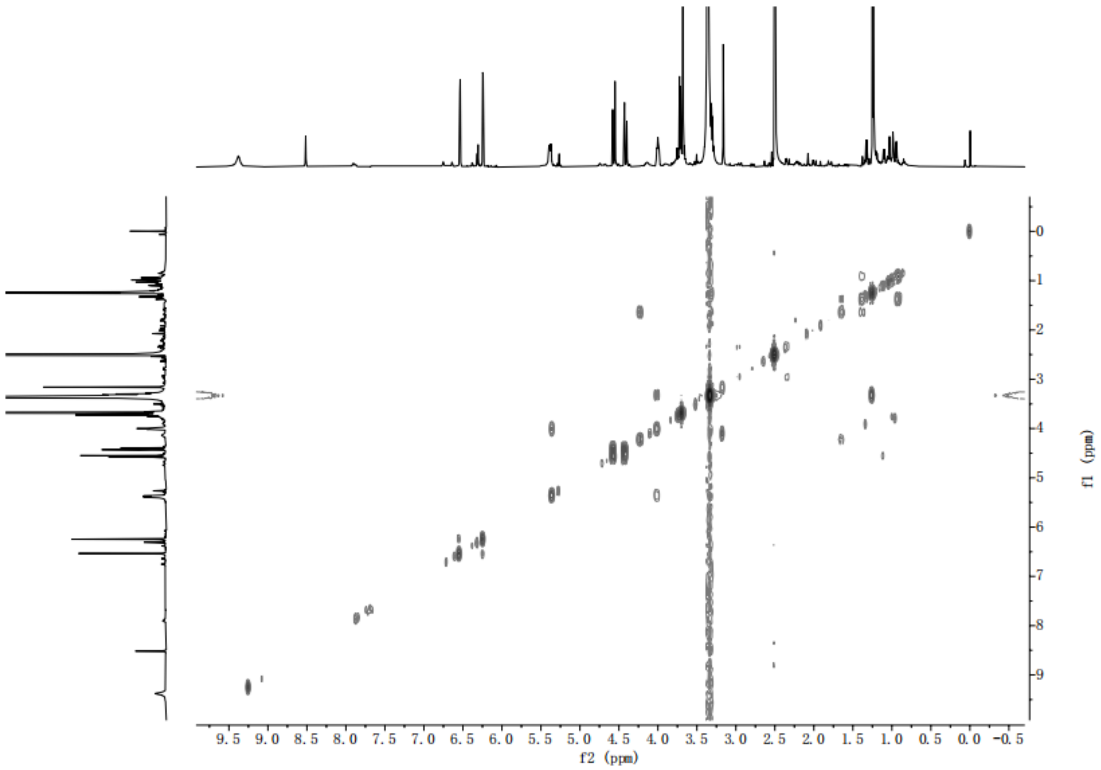

Figure S12. COSY spectrum of compound **2** in DMSO-d_6.

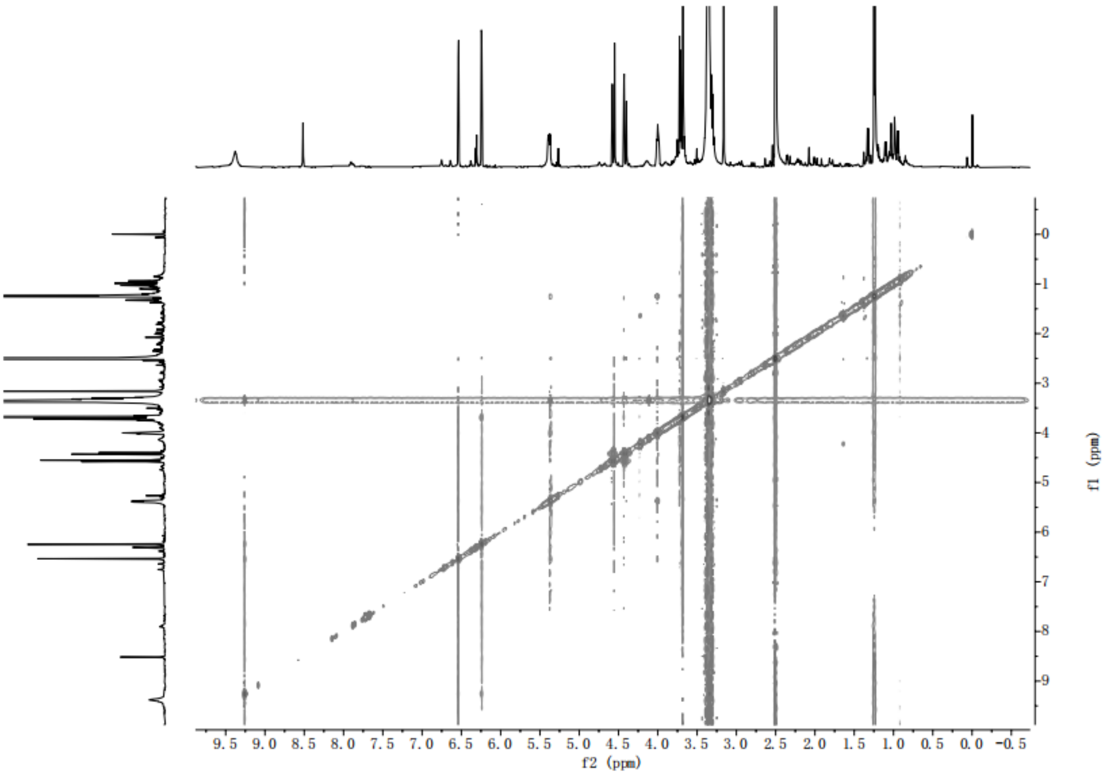

Figure S13. NOESY spectrum of compound **2** in DMSO-d_6.

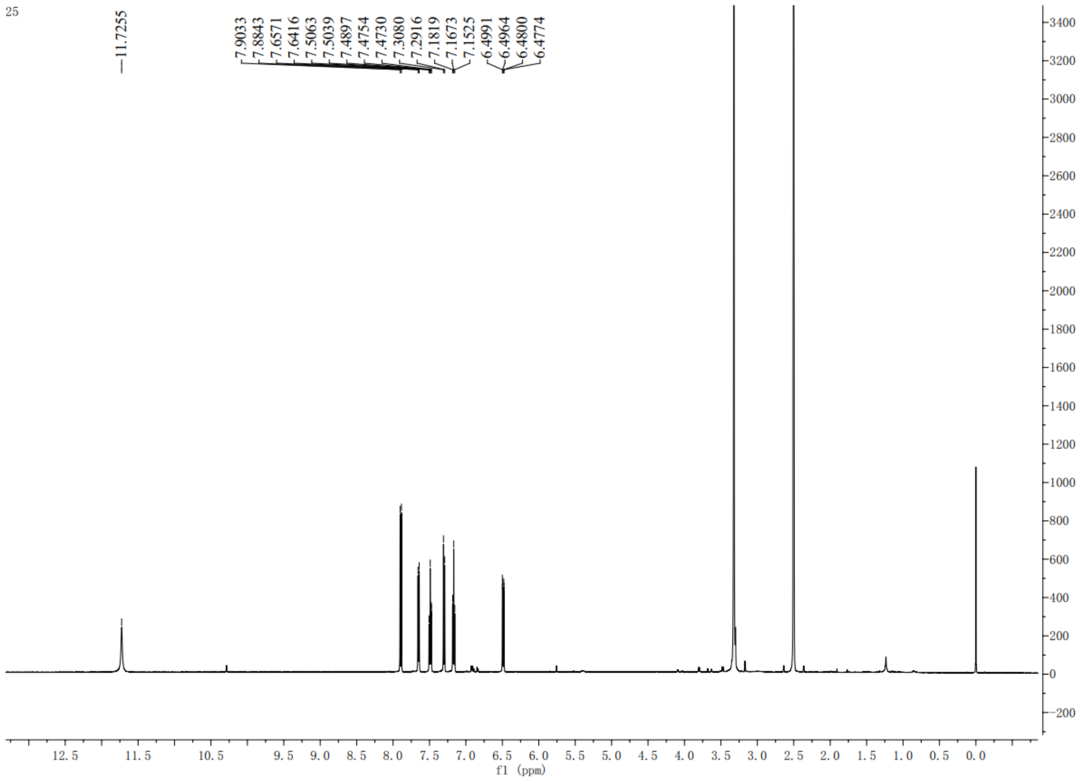

Figure S14. ^{1}H NMR spectrum of compound **3** in DMSO-d_6 (500 MHz).

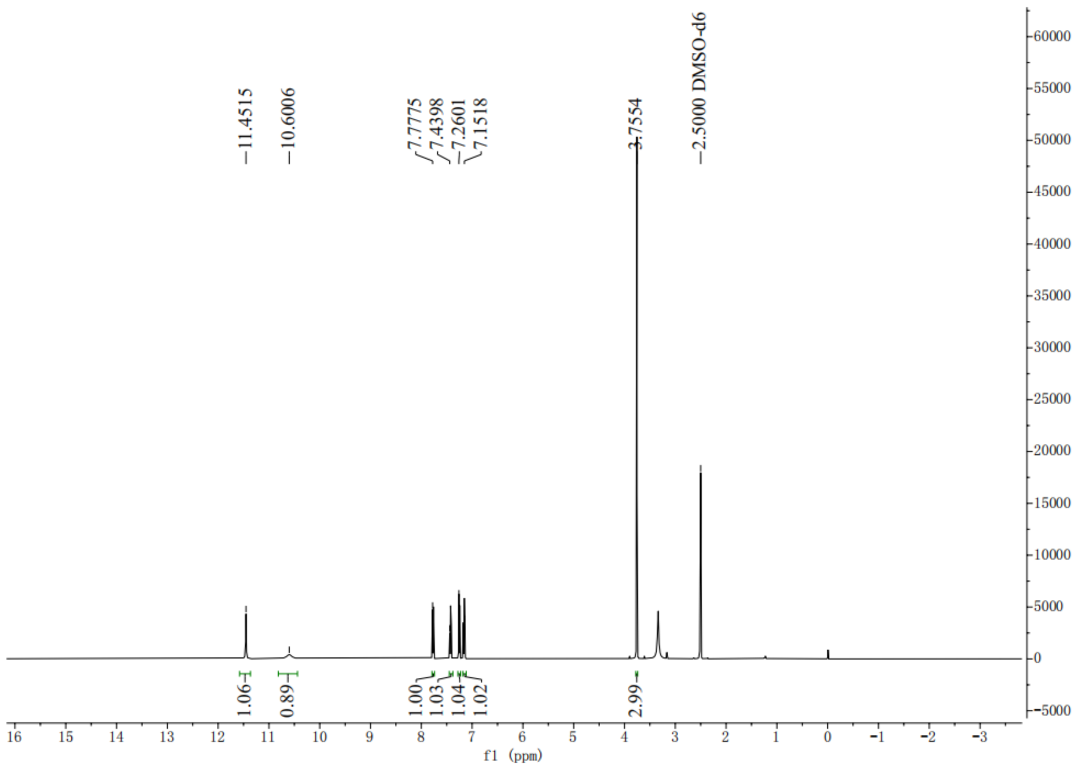

Figure S15. ^{1}H NMR spectrum of compound **4** in DMSO-d_6 (500 MHz).

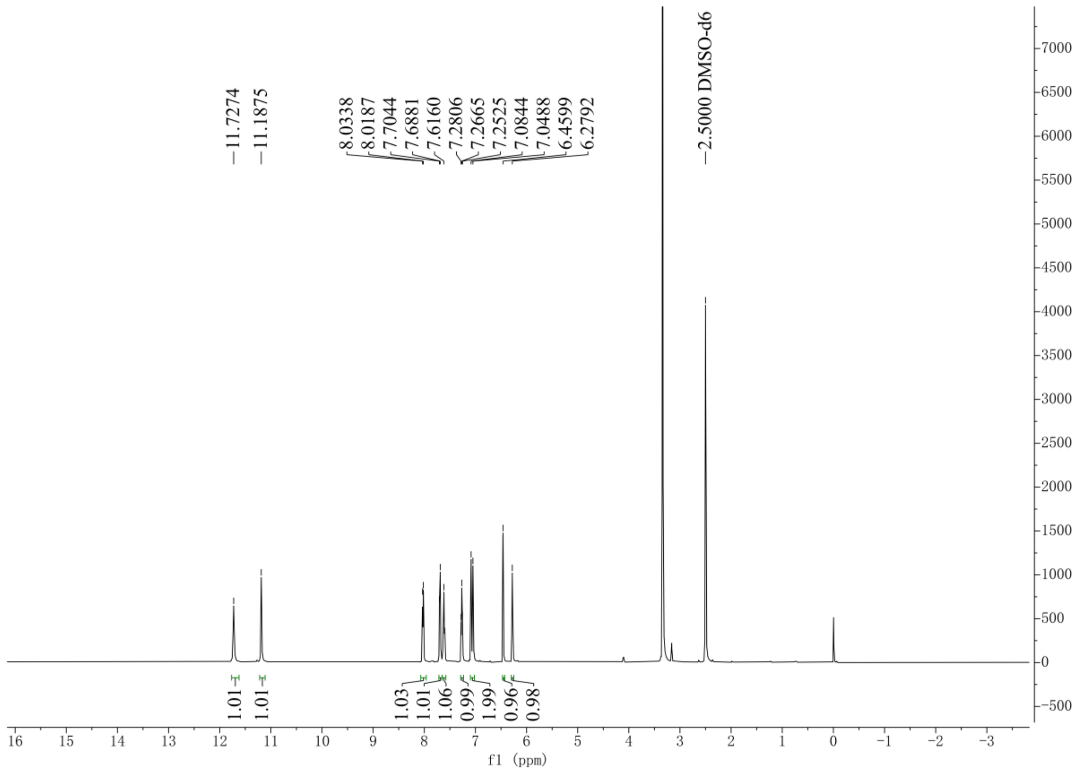

Figure S16. ^{1}H NMR spectrum of compound **5** in DMSO-d_6 (500 MHz).

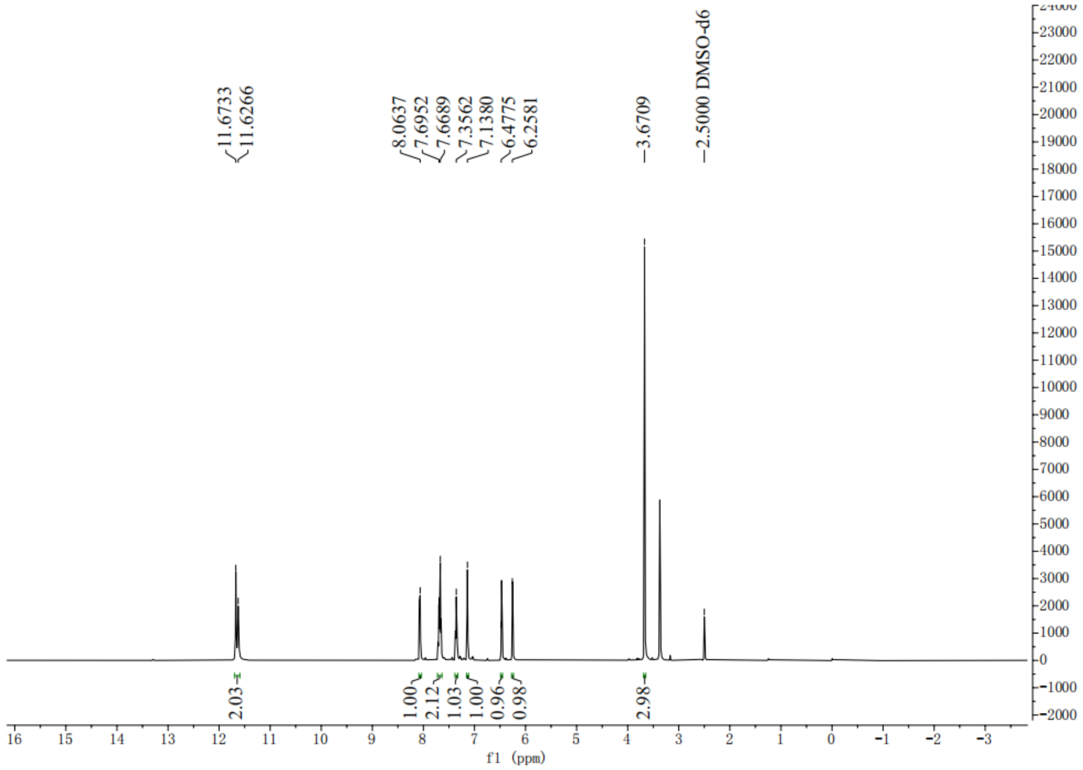

Figure S17. ^{1}H NMR spectrum of compound **6** in DMSO-d_6 (500 MHz).

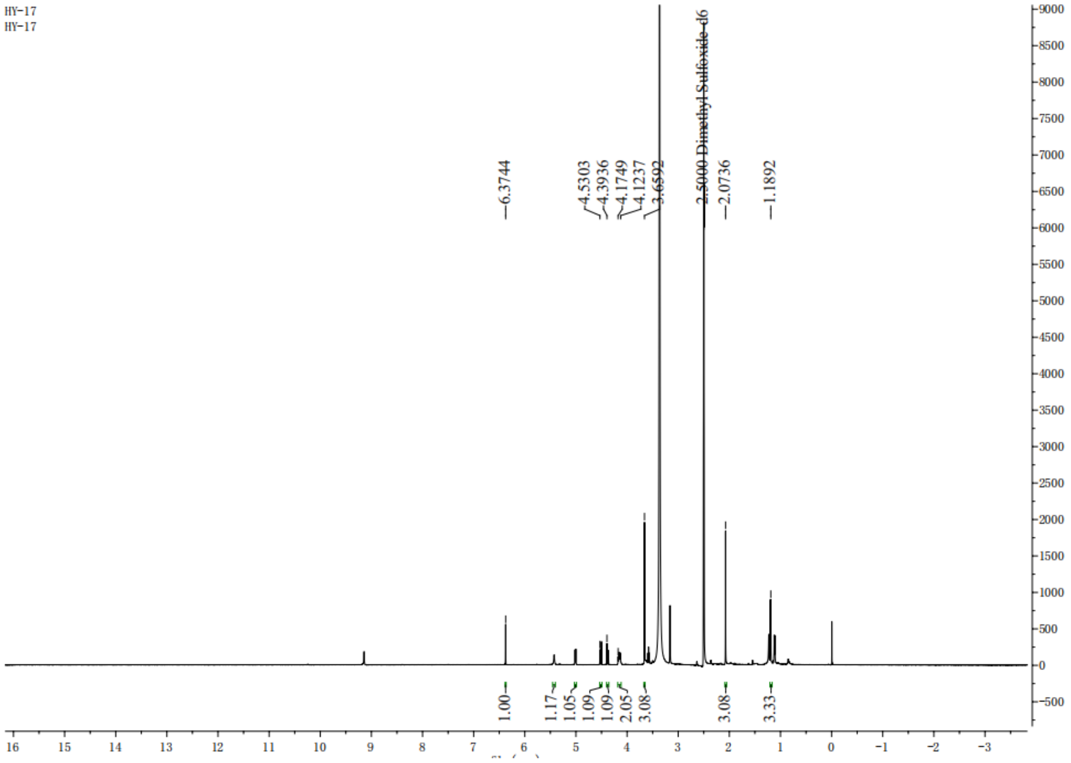

Figure S18. ^{1}H NMR spectrum of compound **7** in DMSO-d_6 (500 MHz).

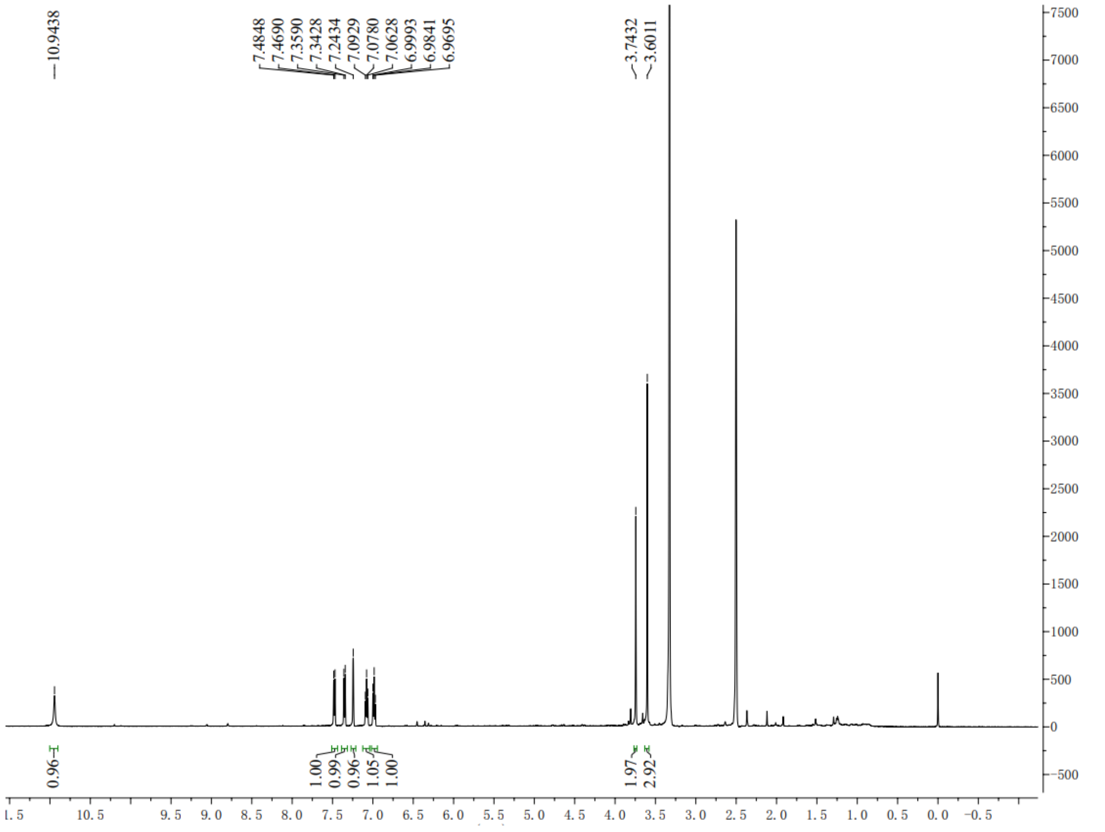

Figure S19. ^{1}H NMR spectrum of compound **8** in DMSO-d_6 (500 MHz).

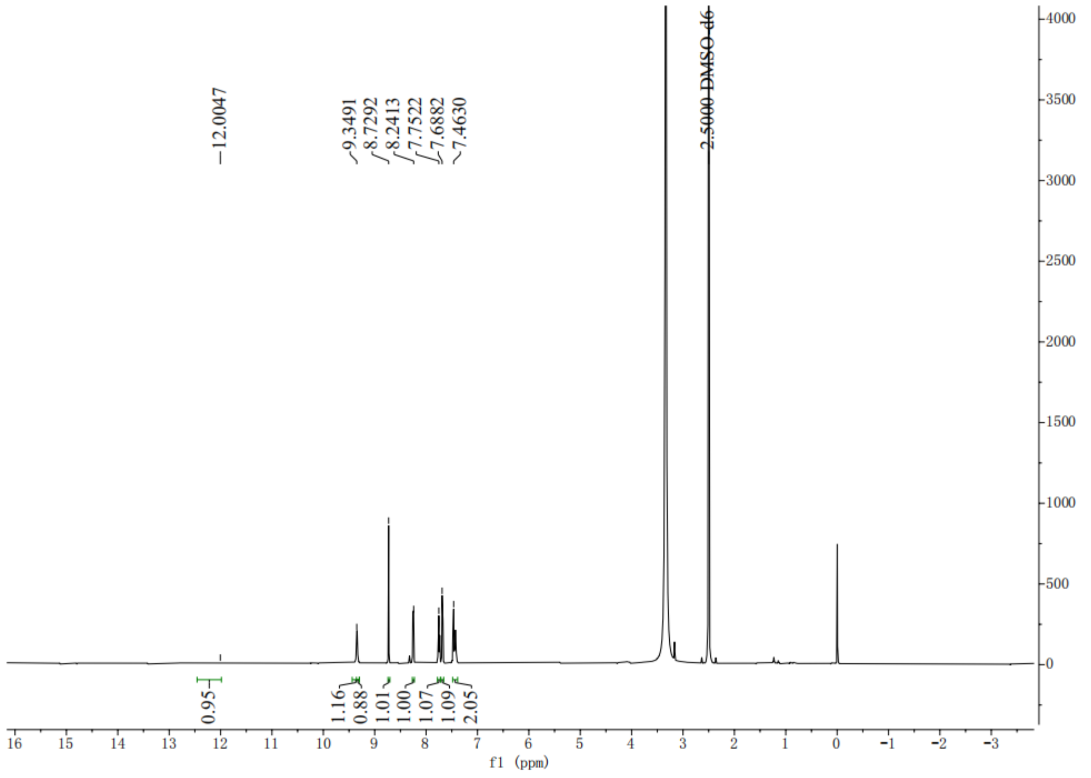

Figure S20. ^{1}H NMR spectrum of compound **9** in DMSO-d_6 (500 MHz).

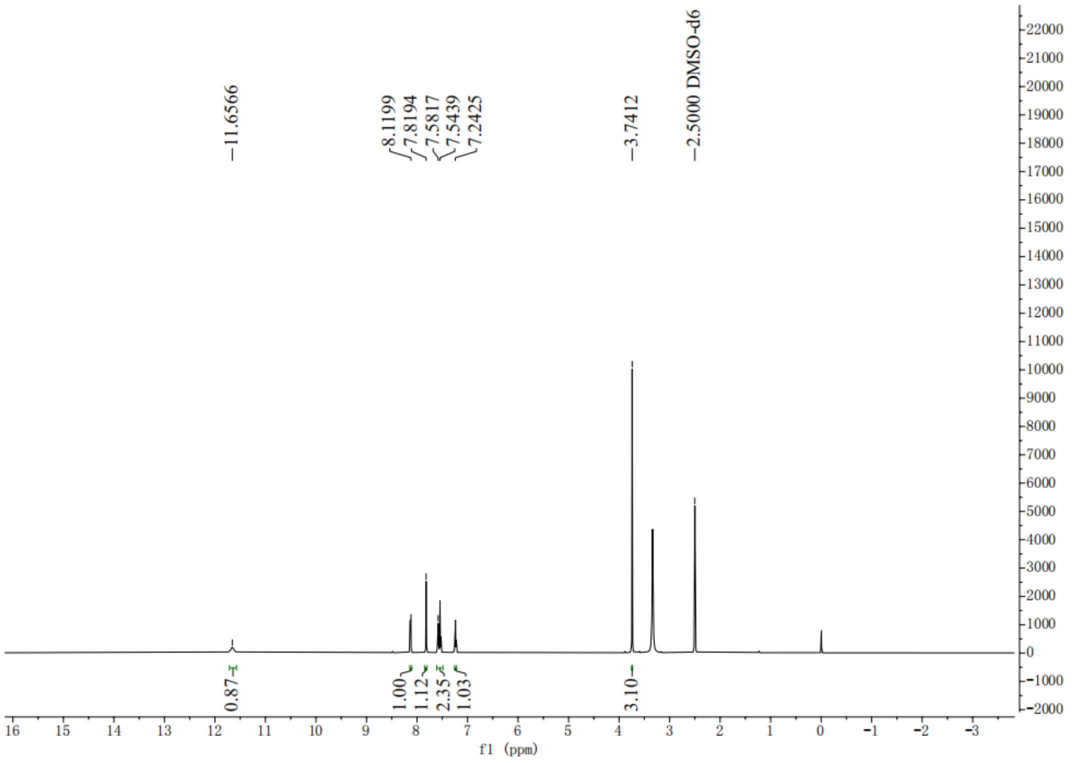

Figure S21. ^{1}H NMR spectrum of compound **10** in DMSO-d_6 (500 MHz).
